# A Comprehensive Review of Bio-Inspired Optimization Algorithms Including Applications in Microelectronics and Nanophotonics

**DOI:** 10.3390/biomimetics8030278

**Published:** 2023-06-28

**Authors:** Zoran Jakšić, Swagata Devi, Olga Jakšić, Koushik Guha

**Affiliations:** 1 Center of Microelectronic Technologies, Institute of Chemistry, Technology and Metallurgy, National Institute of the Republic of Serbia University of Belgrade, 11000 Belgrade, Serbia; olgicajaksic@gmail.com; 2 Department of Electronics and Communication Engineering, B V Raju Institute of Technology Narasapur, Narasapur 502313, India; swagata.devi@bvrit.ac.in; 3 Department of Electronics and Communication Engineering, National Institute of Technology Silchar, Silchar 788010, India; koushik@ece.nits.ac.in

**Keywords:** bio-inspired computation, multiparameter optimization, metaheuristic algorithms, genetic algorithms, artificial intelligence, deep learning, microelectronics, nanoelectronics, nanophotonics, metasurfaces

## Abstract

The application of artificial intelligence in everyday life is becoming all-pervasive and unavoidable. Within that vast field, a special place belongs to biomimetic/bio-inspired algorithms for multiparameter optimization, which find their use in a large number of areas. Novel methods and advances are being published at an accelerated pace. Because of that, in spite of the fact that there are a lot of surveys and reviews in the field, they quickly become dated. Thus, it is of importance to keep pace with the current developments. In this review, we first consider a possible classification of bio-inspired multiparameter optimization methods because papers dedicated to that area are relatively scarce and often contradictory. We proceed by describing in some detail some more prominent approaches, as well as those most recently published. Finally, we consider the use of biomimetic algorithms in two related wide fields, namely microelectronics (including circuit design optimization) and nanophotonics (including inverse design of structures such as photonic crystals, nanoplasmonic configurations and metamaterials). We attempted to keep this broad survey self-contained so it can be of use not only to scholars in the related fields, but also to all those interested in the latest developments in this attractive area.

## 1. Introduction

Nowadays, we are witnessing an enormous popularity and a literal avalanche of bio-inspired algorithms [1] permeating practically all facets of life. Procedures using artificial intelligence (AI) [2] are being built into a vast number of different systems that include Internet search engines [3], cloud computing systems [4], Internet of Things [5], autonomous (self-driving) vehicles [6], AI chips in flagship smartphones [7], expert medical systems [8], robots [9], agriculture [10], architectural designs [11] and data mining [12], to quote just a tiny fragment. AI can chat with humans and even solve problems stated in the common human language [13], generate paintings and other artworks at a textual prompt [14], create music [15], translate between different languages [16], play very complex games and win them [17], etc. AI artworks have been winning art competitions (and creating controversies at that) [15]. Questions are even posed as to whether AI can show its own creativity comparable to that of humans [18]. Many AI functionalities are met in ordinary life, and we may not even recognize them. All of the mentioned applications and many more are exponentially multiplying, becoming more powerful and more spectacular. The possibilities, at least currently, appear endless. Concerns have been raised for possible dangers for humanity as a whole with using AI, and some legislations have already brought laws limiting the allowed performances and uses of artificial intelligence [19].

Scientific breakthroughs behind all of this are nothing short of astounding. Behind each result we see—and behind those that we may not even be aware of—there is an accelerating landslide of publications including at least hundreds of dedicated science journals with a vast number of articles, numerous books and an uncounted number of all possible kinds of intellectual property. Currently, a renaissance of biomimetic computing is in full swing—and it is still spreading, engulfing more and more different areas.

Not all results in the field of biomimetic computing are so spectacularly in the spotlight and followed by hype as those that mimic human behavior or even our creativity. However, maybe the most important achievements are hidden among the results that do not belong to this group. They include handling big data, performing time analysis or performing multi-criteria optimization. Such intelligent algorithms that are mostly “invisible” to the eyes of the general public are causing a silent revolution not only in engineering, physics, chemistry, medicine, healthcare and life sciences, but also in economics, finance, business, cybersecurity, language processing and many more fields.

Our attention in this text is dedicated to bio-inspired optimization algorithms. They are extremely versatile and convenient for complex optimization problems. The result of such wide applicability is their overwhelming presence in diverse fields—there are practically no areas of human interest where they do not appear. As an illustration of their ubiquity, we mention here just some selected fields where their applications have been reported. They encompass various branches of engineering, including mechanical engineering (automotive [20,21], aerospace [22], fluid dynamics [23], thermal engineering [24], automation [25], robotics [26], mechatronics [27], MEMS [28,29], etc.), electrical engineering [30] (including power engineering [31], electronics [32], microelectronics [33] and nanoelectronics [33], control engineering [34], renewable energy [35], biomedical engineering [36], telecommunications [36], signal processing [37]), geometrical optics [38], photonics [39], nanophotonics and nanoplasmonics [40], image processing [41] including pattern recognition [42], computing [30], [43], networking (computer networks [44] including Internet and Intranet [45], social networks [46], networks on a chip [47], optical networks [48], cellular (mobile) networks [49], wireless sensor networks [50], Internet of things [51], etc.), data clustering and mining [52], civil engineering [53,54], architectural design [55], urban engineering [56], smart cities [57], traffic control and engineering [58], biomedicine and healthcare [59,60], pharmacy [61,62], bioinformatics [63], genomics [64], computational biology [60], environmental pollution control [65] and computational chemistry [66]. Other optimization fields where biomimetic algorithms find application include transportation and logistics [67], industrial production [68], manufacturing including production planning, supply chains, resource allocation and management [69], food production and processing [70], agriculture [71], financial markets [72] including stock market prediction [73], as well as cryptocurrencies and blockchain technology [74], and even such seemingly unlikely fields as language processing and sentiment analysis [75]. The cited applications are just a tip of an iceberg, and there is a vast number of other uses not even mentioned here.

According to the 1997 paper by Wolpert and Macready titled “No free lunch theorems for optimization”, if an algorithm finds the best solutions in one field, chances are that it will not perform so well in others [76]. This means that no algorithm will always find the optimum solutions in all fields. Because of that, there is an enormous number of different algorithms and algorithm modifications or improvements that excel in some areas, and some of them even in many, but each one of them will have its own peculiarities, advantages and disadvantages. Thus, a logical consequence of such a situation is the existence of a large number of review papers attempting to sort out the state of affairs among the numerous different algorithms. The situation is not facilitated by the fact that some of the metaheuristic algorithms actually overlap with others and are similar, or in some cases are literally identical among themselves, the main differences being in the algorithm names [77]. In addition, some algorithms that had been very popular some years ago fell out of use while others rose to fame. Due to the mentioned reasoning, there is a constant need for updated reviews. Another problem is related to the enormous extent of the field. While excellent and exhaustive in-depth critical reviews naturally do appear, the majority only cover some particular subjects, out of the sheer impossibility of encompassing everything, while many do not even attempt to achieve a comprehensive coverage and focus on assorted bits instead.

To render this work, we analyzed 108 review papers and monographs on bio-inspired optimization algorithms (not all of which are cited here) plus numerous contributed research articles which contain review sections, each typically a few pages long. We believe we created a unique survey that covers a number of topics that none of the above-mentioned sources considered and which, to the best of our knowledge, cannot be found in a single place. In other words, we attempted to offer a synthesis of different subjects that contains updated information and offers as wide an overview as we were able to create. With this work, we tried to write a self-contained and comprehensive material covering the main fields from among multitudinous and often redundant (and in some cases even conflicting) bio-inspired optimization algorithms in a form accessible to as wide a multidisciplinary scientific audience as possible.

We attempted to include some of the most recent results (years of publication 2022 or 2023) that could not have possibly been mentioned in a vast majority of the previous review papers due to the simple fact that these results did not exist at the time. Obviously, such publications were not present for a sufficient time to allow a confident measure of the degree of their acceptance by the scientific community. Thus, our choice had to be partly subjective. We also took care to include the topics that in our opinion are of high importance now and for which we anticipate even higher impact in the future (some examples being multi-objective and hybrid optimization algorithms). At the same time, we strived not to omit older but still significant and widely used methods. We are well aware that in today’s rapidly expanding and branching field of biomimetic AI optimization algorithms, we may have overlooked and omitted some important sources, but this is almost inevitable in the current environment.

Another contribution of this text is related to the systematization and taxonomy of some topics in the field. There are contradictory reports in the literature on classification and even on some definitions, and we tried to present our point of view on it. We offered some modifications to the classification that we hope could serve at least a bit better than some of those previously published. We also attempted to clarify a few conflicting pieces of information from prior works.

Further, as an example, we dedicated a part of our review to two partially interconnected fields, namely microelectronics and nanophotonics. We are unaware of encountering that combination in a single comprehensive text, and even less one written in this manner. The importance of this inclusion is also reflected in the fact that optimization algorithms are rarely included in the typical curricula of the researchers in these two fields and are mostly related to the profiles of mathematicians and computer scientists.

We made efforts to keep the writing style as simple and clear as possible, yet exact and with correct nomenclature. At the same time, we tried to avoid excessive in-depth handling of any narrowly specialized field. This was done to ensure the usefulness of the manuscript to a range of researchers at different levels, from beginners to experts in the field, as well as to casual readers, i.e., to make it accessible to the widest circle of scientific audience. Our hope was that the present work could become handy as a kind of user-friendly one-stop all-purpose manual and a comprehensive overview of the main points to ensure simpler navigation through the enormously vast body of literature, especially for such a multidisciplinary-oriented audience as that gathered around the *Biomimetics* journal.

The landscape of biomimetic optimization algorithms is rapidly evolving, and new advances are being introduced daily. This means that any reviews of the state of the art will necessarily become dated relatively quickly. Thus, it is essential to bring as updated information about the existing techniques as possible. This broad survey, while the authors are well aware of the enormity of the task and the inevitable shortcomings and incompleteness of the work that stem from the pure impossibility of being all-encompassing, strives to offer its modest contribution to staying updated at least for the time being. This work may be thus seen as a partial snapshot of an explosively spreading and evolving field.

**Figure 1:**
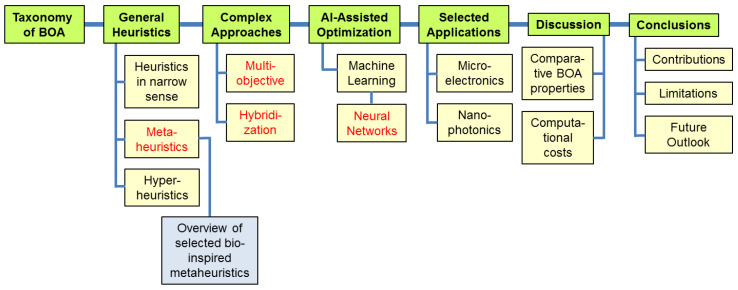
Schematic overview of the topics presented in this broad survey of bio-inspired optimization algorithms (BOAs).

The manuscript is structured as follows: Section 2 presents a possible taxonomy of different bio-inspired optimization algorithms and considers the redundancy of some of the existing procedures. The following sections briefly present some of the most important and well known ones, such as heuristic procedures including biology-based metaheuristic algorithms and hyper-heuristics, neural networks and hybrid methods. As an illustration, a section is dedicated to the advances in the application of bio-inspired multi-criteria optimization in microelectronics, and another section is dedicated to the recent applications in nanooptics and nanophotonics. These are followed by some conclusions and an outlook. Due to their relative complexity, an overview of the topics presented in this work is schematically shown in Figure 1.

## 2. A Possible Taxonomy of Bio-Inspired Algorithms

In this section, we present one possible hierarchical classification of bio-inspired algorithms. The consideration has been made without taking into account any specific targeted applications of the algorithms. Generally, taxonomies of bio-inspired algorithms are relatively rarely considered in the literature. The majority of papers simply skip the topic altogether or handle it casually, presenting only the methods that are of immediate interest to the subject of the paper or, even more often, giving only a partial and non-systematic picture and denoting it as a classification. This is not to say that exhaustive and systematic papers on the subject do not exist. However, it appears that no consensus has been reached about the taxonomy of at least some bio-inspired algorithms yet. 

Some quality papers dedicated to the topic and published within the last few years include [78,79,80]. In this article, we present our view of the subject that includes many elements of the previously proposed classifications, but also incorporates novel ones, as well as updated information on some approaches proposed within the last few years, which could not be included previously since they were presented after the quoted papers appeared. We do not claim the generality of our taxonomy, although we did try to incorporate as much available data as we were able.

A problem when attempting to define a categorization in this field is that some approaches, although having different names, actually present algorithms very similar or even basically identical to those previously published. Often they offer only incremental advances, such as somewhat better results at benchmarks of precision or computing speed. This is a very slippery ground, however, since according to the previously mentioned No Free Lunch Theorem [76], no algorithm is convenient for all purposes, and while one of them may offer a fast and accurate solution to one class of optimization problems, there is no guarantee that it will not perform drastically worse with other problems, become stuck in a local optimum, never even reaching a global optimum, or even fail completely to give a meaningful solution. For this reason, it is very difficult to decide which procedures merit inclusion in the classification and which do not.

A number of benchmarks have been proposed to compare different optimization procedures, and the most recent publications in the field use them to prove the qualities and advantages of their proposals over the competing ones. A systematic review of methods to compare the performance of different algorithms has been published by Beiranvand, Hare and Lucet [81]. A more recent consideration of that kind dedicated to metaheuristics has been presented by Halim, Ismail and Das [82], who offered an exhaustive and systematic review of measures for determining the efficiency and the effectiveness of optimization algorithms. A benchmarking process for five global approaches for nanooptics optimization has been described by Scheider et al. [83].

**Figure 2:**
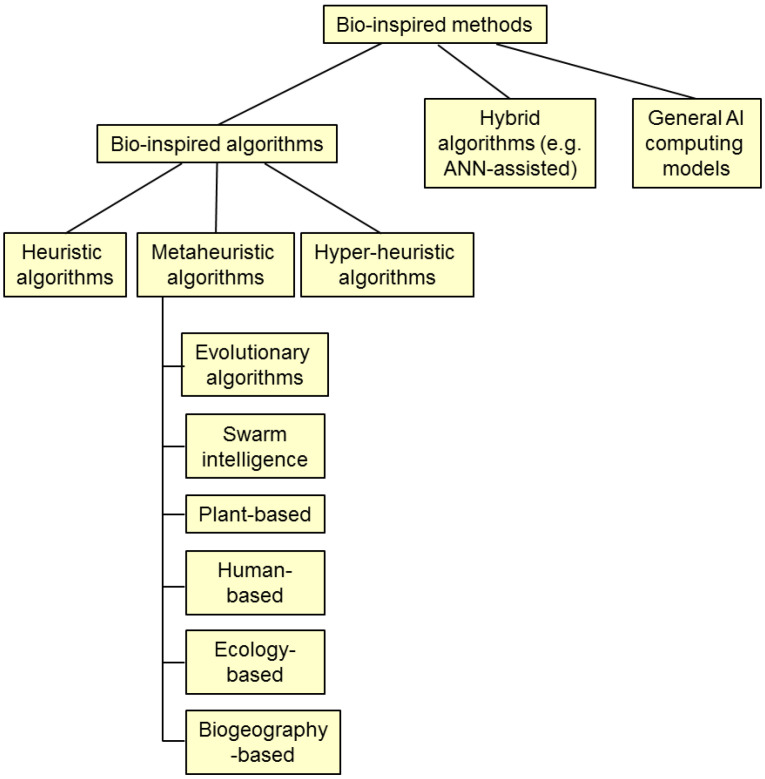
Possible classification of bio-inspired optimization methods.

One can find various taxonomy proposals in the literature, each with its own merits and disadvantages. Figure 2 represents the scheme of a possible classification of bio-inspired optimization methods.

## 3. Heuristics

Heuristics can be briefly described as problem solving through approximate algorithms. The word stems from the Ancient Greek εὑρίσκω (meaning “to discover”). It includes approaches that do not mandatorily result in an optimum solution and are actually imperfect, yet are adequate for attaining a “workable” solution, i.e., a sufficiently good one that will probably be useful and accurate enough for a majority of cases. On the other hand, they may not work in certain cases, or may consistently introduce systematic errors in others. The methods used include pragmatic trade-offs, rules of thumb (use of approximations based on prior knowledge in similar situations), a trial and error approach, the process of elimination, guesswork (“educated guesses”) and acceptable/satisfactory approximations. The main benefit is that heuristic approaches usually have vastly lower computational cost, and their main deficiencies are that they are usually dependent on a particular problem (i.e., not generally applicable in all situations) and their accuracy may be quite low in certain cases, while inherently they do not offer a way to estimate that accuracy.

Heuristic approaches include common heuristic algorithms, metaheuristic algorithms and hyper-heuristic algorithms. All of these approaches are considered to represent the foundations of AI.

### “Basic” Heuristic Algorithms

The heuristic algorithms represent the oldest approximate approach to optimization problems, from which metaheuristics and hyper-heuristics evolved. They include a number of approximate goal attainment methods. While there is no universally accepted taxonomy of common heuristic algorithms, a possible classification is presented in Table 1. Metaheuristic and hyper-heuristic algorithms are not included in this subsection, since these are covered separately in the next two sections. This is a short overview only, presented for the sake of generality, since the quoted algorithms are mostly unrelated to bio-inspired methods. The comprehensiveness of the table is not claimed, and some quoted methods may overlap more or less, thus appearing in multiple categories at the same time.

## 4. Metaheuristics

Metaheuristics represent a conceptual generalization and enhancement of the heuristic approach. While the literature usually does not appear to provide a clear and consistent definition of metaheuristics and there seems not to be a consensus about it, it does offer various descriptions, among which is that metaheuristic algorithms represent iterative global optimization methods that make use of some underlying heuristics by making an intelligent combination of various higher-level strategies for exploring the search space, seeking to avoid local optima and to find an approximate solution for the global optimum [94,95]. The mentioned approaches are typically inspired by natural phenomena and mimic them. These phenomena may be for instance animal or human collective behavior, physiological processes or plant properties, but they also include some non-biological processes such as physical, astrophysical or chemical phenomena and mathematical procedures [96] (these non-biomimetic algorithms are not covered by this treatise).

The methods in metaheuristics are sometimes denoted as metaphor-based since their naming and design are more or less inspired by actual biological and other processes. The metaheuristics are the best known, most popular and by far most often applied among the heuristic methods, and the papers dealing with them are the most numerous group of publications on nature-based optimization algorithms.

Many new procedures that belong to this group are constantly being proposed, almost on a daily basis. A paper by Ma et al. [97] presented an exhaustive list of more than 500 metaphor-based metaheuristic algorithms and their benchmark basis. While many of the proposed methods simply represent reiteration or sometimes even literal renaming of known methods, some newly described approaches do show relevance and usability and introduce new levels of sophistication and performance. As mentioned before in this text, the existing tsunami of metaphor-based algorithms has been heavily criticized by some researchers, who have been arguing that the approach is fundamentally flawed and that a new taxonomy should be introduced since it would expose the essential similarity among many of the newly proposed methods [98].

Swarm intelligence (SI) algorithms (mostly based on the collective behavior of animals) are by far the largest of the metaheuristic procedures and biomimetic computation approaches generally. They encompass the largest part of bio-inspired algorithms, amounting to about 67.12% of all of them. The article [80] calculates that about 49% of all nature-based methods belong to this class; however, if we do not take into account those of non-biological origin, a simple recalculation brings us to a percentage of more than 67%.

### 4.1. Evolutionary Algorithms (EAs)

The group of evolutionary algorithms (EAs) includes different population-based metaheuristic optimization algorithms. They are inspired by the processes of Darwinian evolution of species, including the procreation of offspring, genetic mutations, recombination and natural selection. They are not related to any particular specificities of any concrete optimization problem and thus are applicable to a very wide variety of different scenarios. They have a predefined objective (fitness) function determining the desired quality of the solution, and the candidate solutions are individuals in the population. There are numerous metaheuristic procedures that belong to the EA group.

Table 2 shows a few selected basic EAs. It quotes the name of the algorithm first, then its standard abbreviation, the names of its proposers or key proponents and the year when the proposal was first presented (or the main popularization event occurred), and finally an important reference related to the topic (either the original publication that proposed the algorithm or a comprehensive survey or review). In the subdivisions following Table 2, short descriptions of the selected algorithms are given.

#### 4.1.1. Genetic Algorithms (GAs)

Genetic algorithms (GAs) (Holland, 1975) [102] are a class of optimization algorithms belonging to the evolution-based bio-inspired metaheuristics that mimic the Darwinian process of natural selection and genetic evolution.

A GA is a population-based optimization algorithm. The potential solutions to an optimization problem are represented as individuals within a population. These individuals are denoted as a genotype of chromosomes and represent potential solutions to the optimization problem, regardless of the field to which they belong (in our case microelectronics and nanophotonics). Their data are encoded as a string of binary digits that can be further manipulated and processed.

An initial population of chromosomes/individuals is randomly generated, based on the properties of the optimization problem to be solved by the GA. The fittest chromosomes/individuals are then selected as a subset of the chromosome population. The strategies for the assessment of the fittest individual can be rank-based, or some kind of procedure for the choice of the fittest may be applied. These procedures include roulette wheel selection and tournament selection. In this manner, candidate members of the population are compared among themselves, and the fittest ones are chosen as the parents for the next generation. Crossover and mutation operators are applied to them in each iterative cycle to ensure variety, and the process is repeated until the optimum or near-optimum solution is found or, alternatively, until the number of iterations exceeds the predefined value. In this manner, the quality of solutions gradually improves over successive generations. Genetic operations guard the diversity of the solutions. In this way, the algorithm is able to investigate different areas of the search space and reach the global optimum, avoiding being trapped in local optima.

**Figure 3:**
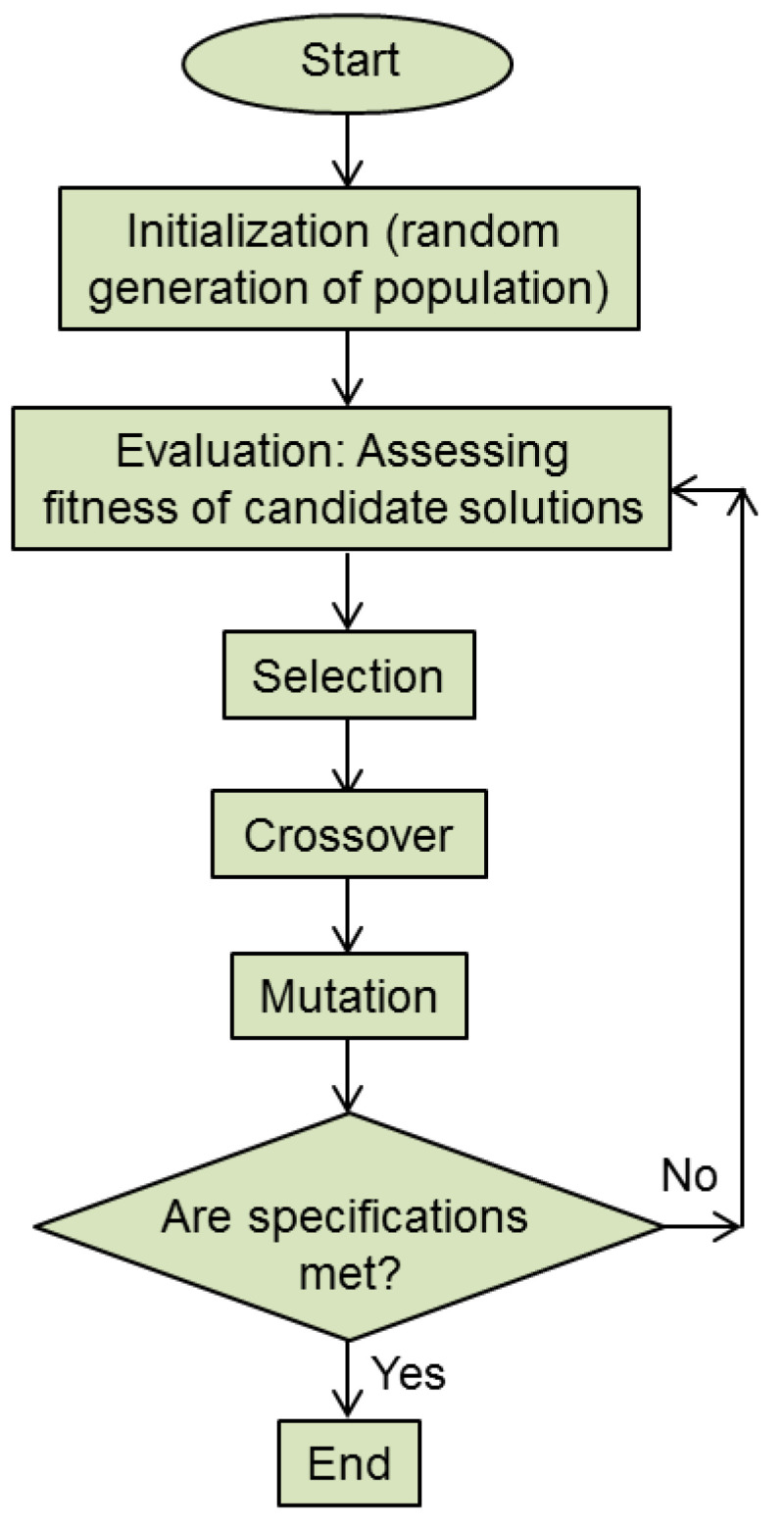
Genetic algorithm flowchart.

Figure 3 shows a simplified flowchart of a genetic algorithm. The important Darwinian steps (selection, crossover and mutation) are performed after each evaluation is performed among the members of the subset of the fittest. This procedure is repeated iteratively, each iteration representing a single generation of the genetic evolutionary process.

#### 4.1.2. Memetic Algorithms (MAs)

Similarly to GAs, memetic algorithms (MAs) [100] are optimization algorithms belonging to the evolutionary bio-inspired algorithms, but they combine the Darwinian process of natural selection and evolution with the behavior of a meme, as conceptualized by Dawkins [103]. The Darwinian part is reflected in the application of the principles of evolutionary computation, i.e., population-based evolutionary processes, while the meme represents local search operations that enhance the exploration performance of the algorithm. In other words, memetic algorithms represent an extension/enhancement of the genetic algorithm that improves the convergence speed and the overall search quality. The local search component (meme) utilizes the promising regions of the search space through the iterative improvement of individual solutions.

Typically, memetic algorithms consist of five steps:

1. Initializing the Population: Random generation of candidate solutions.
2. Evaluation: The fitness of each candidate solution is assessed according to the problem’s fitness criterion (objective function).
3. Evolutionary Process: Genetic operators are applied (selection, crossover and mutation) according to the standard evolutionary algorithm rules; thus, the population evolves through generations.
4. Local Search: In addition to the evolutionary processes, local search techniques (memes) are applied to refine or improve individual solutions. This local search often utilizes problem-specific knowledge or heuristics to locally explore the solution space more accurately.
5. End: The algorithm terminates when the ending criterion is met—achieving a satisfactory solution or reaching the maximum set number of iterations.

**Figure 4:**
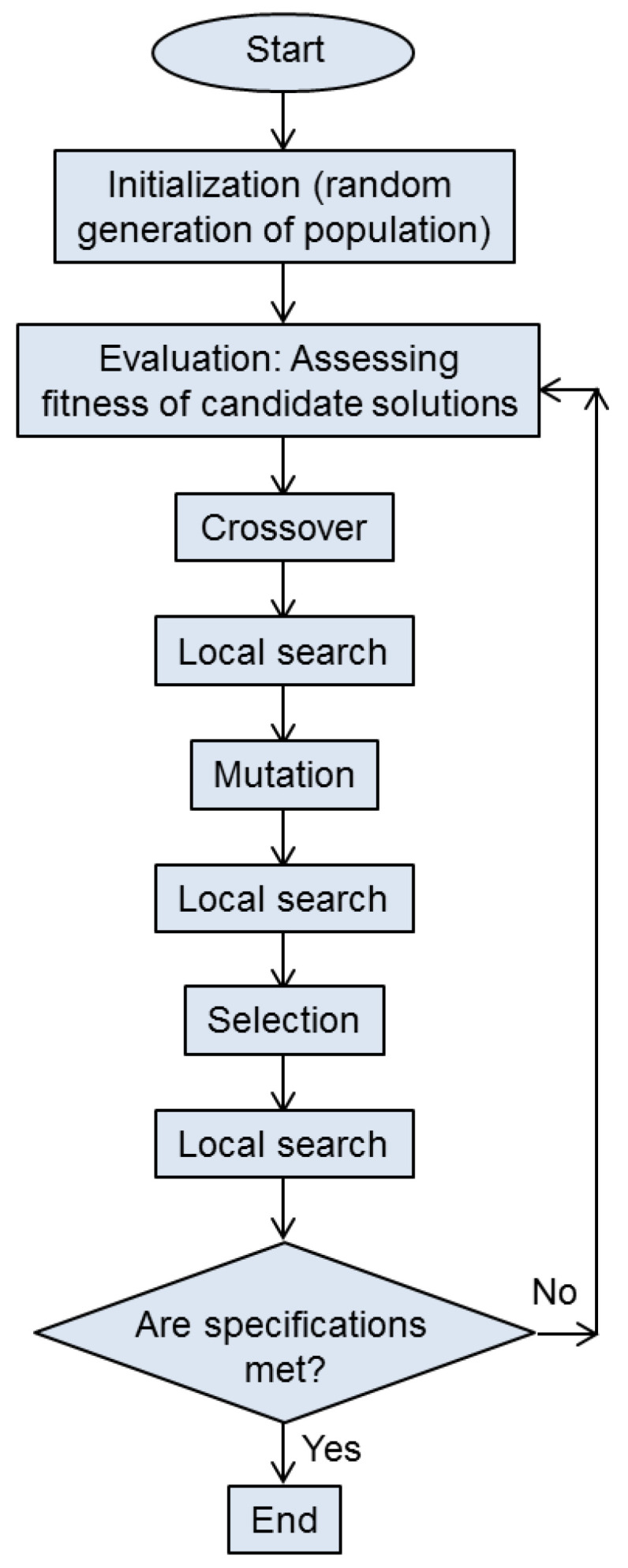
Memetic algorithm flowchart.

A possible flowchart of a memetic algorithm is shown in Figure 4.

#### 4.1.3. Differential Evolution (DE)

The differential evolution algorithm [101] is probably the most frequently published and analyzed of all bio-inspired algorithms, having appeared as the main topic of more than 86,500 publications as of May 2023 (see Figure 1 in [97]). It includes a population of candidate solutions (also denoted as vectors or agents) and objective vectors. Basically, this algorithm is generally similar to a genetic algorithm; however, there are significant differences. The main dissimilarity is related to the choice of parents for offspring. While in a genetic algorithm, the candidates are compared among themselves, usually through some selection procedure (choice of the fittest, roulette wheel or tournament selection), here they are selected by comparison of the trial agents (candidate solutions) with the objective vectors (the target).

Mutations proceed by forming a mutant vector by randomly picking two individuals from the existing population (base vectors), calculating their difference and adding its weighted value to the objective (target) vector. This is denoted as differential mutation. The mutant vectors are recombined with the target vectors, and the results of this operation are trial vectors. The recombination or crossover proceeds with each component of the trial vector being either chosen from the mutant vector or from the target vector. This is accomplished according to the crossover rate (recombination rate), which represents a predefined probability. In this way, the Darwinian survival of the fittest is replaced by the combination with the target vector. The trial vectors are then compared to target vectors according to the fitness evaluation. If a trial vector represents an improvement over the corresponding target vector, it replaces the target vector. If not, the target vector is not replaced. In this way, the selection is accomplished and evolution continues.

**Figure 5:**
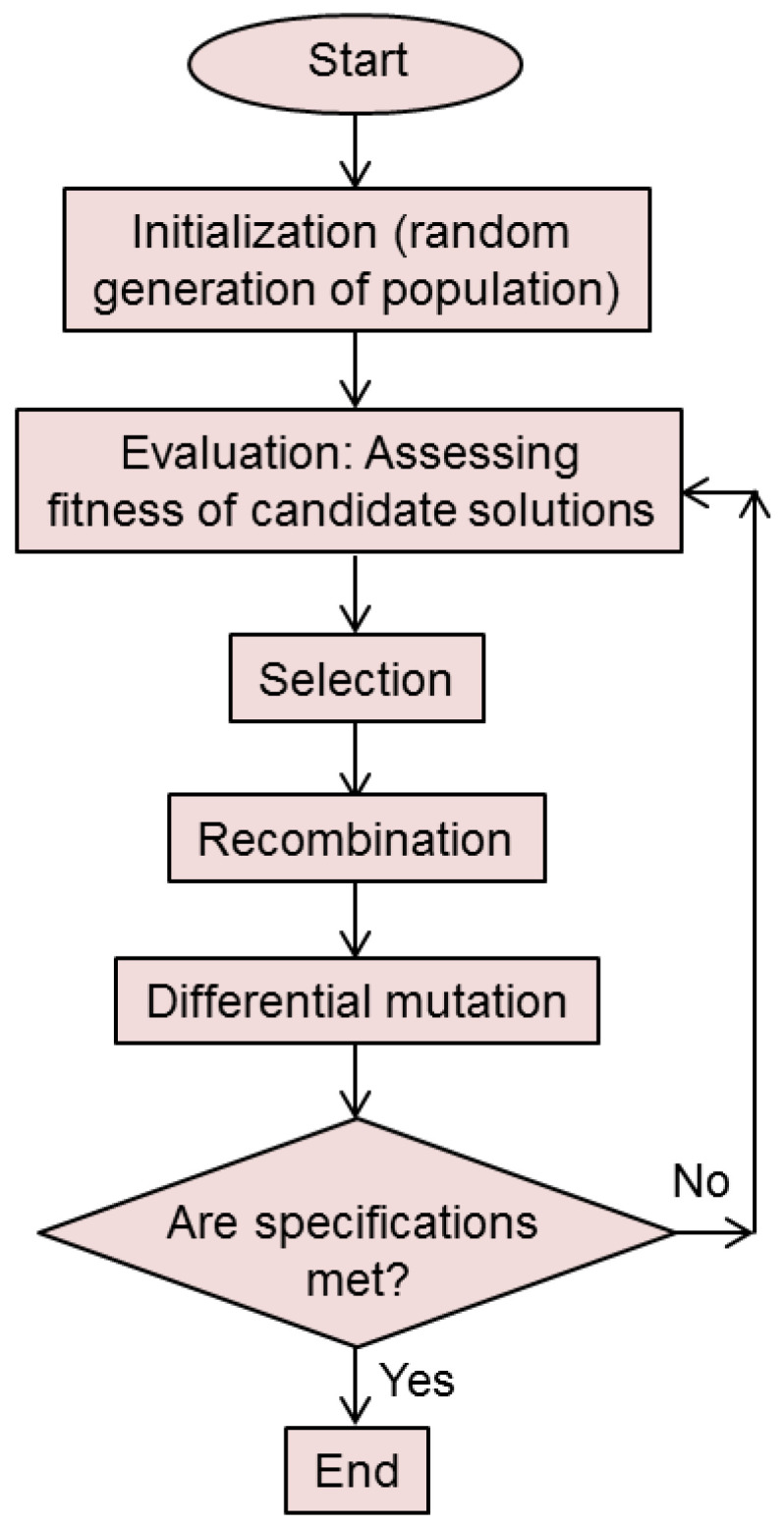
Flowchart of differential evolution algorithm.

Figure 5 shows a flowchart of the DE algorithm. The iterative procedure proceeds until the set objective is reached or the maximum number of iterations is exceeded.

The DE method is extremely popular due to its advantages such as simplicity, ability to work with difficult functions and robustness. Since DE does not require its functions to be differentiable because it does not need any calculations of gradients, it is convenient for discontinuous problems, as well as time-variable problems, cases with high levels of noise and other optimization tasks related to complex problems. All of these properties make it suitable for a wide range of different complex problems, which is the explanation for its overwhelming popularity and widespread use.

### 4.2. Swarm Intelligence (SI) Algorithms

Swarm intelligence algorithms are a huge group of nature-inspired metaheuristic methods dealing with complex optimization problems for which exact mathematical or traditional approaches are difficult or even impossible to implement. They are based on the social behavior of large collectives of animals (flocks of birds, schools of fish, swarms of insects, herds of large mammals) and their ways of attaining their goals. This is also the main part of our taxonomy consideration, and some of the most-used and best-known algorithms belong to it.

In the following part, some illustrative examples of swarm intelligence algorithms are briefly outlined. The criterion of their inclusion was either their acceptance by the scientific and engineering community, as seen through the number of publications investigating a particular algorithm and the number of citations, or their novelty—some quite recent algorithms are also presented, and the inclusion criterion was the number of citations (if some very recently proposed methods gathered a relatively large number of citations in a short time, they probably merit inclusion). The authors of this text are aware that such an approach may have inherent issues with subjectivity and the choice of criteria. In addition, the number of existing swarm intelligence methods is overwhelming (and growing daily), and it is difficult to estimate if some particular omitted cases were to be preferred over those chosen. Thus, some important methods may have been skipped, while some other less important ones may have been taken into account. We strived to keep such situations at a minimum.

Table 3 shows some selected swarm intelligence algorithms. The Table 1 description is valid for its contents. In the subdivisions following, Table 3 some selected algorithms quoted in it are described in more detail.

#### 4.2.1. Particle Swarm Optimization (PSO)

PSO is the most popular swarm intelligence (SI) algorithm, with about 66,000 publications as of May 2023 [97]. In the same way as the SI approach in general, PSO is inspired by the collective behavior of large groups of social animals (insects, fish, birds, mammals). It is a population-based metaheuristic algorithm, especially convenient for continuous search spaces.

Each particular social animal in the swarm is regarded as a single “particle” or point in the search space, defined by its location and velocity, which approaches the ideal solution according to an objective function (for a social animal, that solution can be the food location, mating pair location or another goal). Every particle typically changes its velocity *v* toward its target by using its own experience of the position, plus the experience of its neighbors (the location of the particles in the nearby surroundings), the location data of all the particles searching for the solution with a predefined inertia *w*. Each particle’s motion is defined by four variables:

1. Its current position in the search space;
2. Its best position in the past—past best (Pbest);
3. The best position in its direct proximity—local best (Lbest);
4. The ideal position for all particles combined—global best (Gbest).

Using these parameters, every particle will update its data and will follow the relation

Current [*i*] = current [*i*] + *v* [*i*] (1)

where *i* denotes the number of the concrete particle in the swarm. The velocity of the particle is updated according to

**Figure 6:**
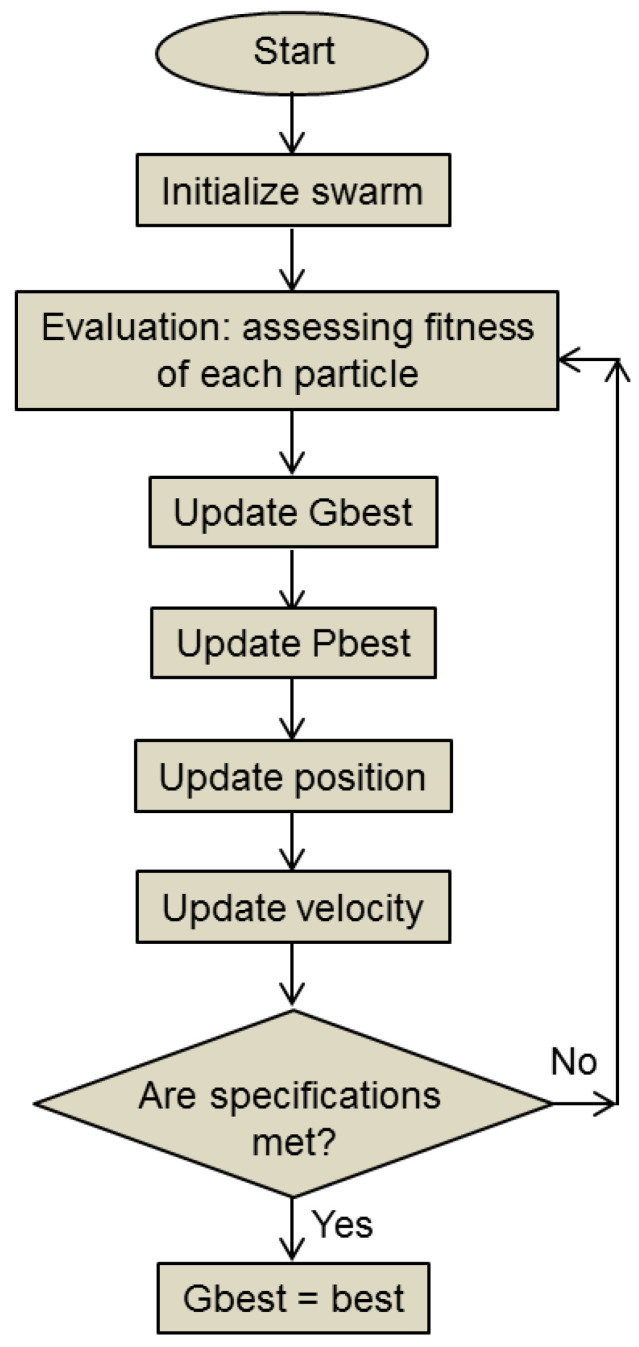
Flowchart of basic PSO algorithm.

*v_i_* = 𝑤 × *v_i_* + 𝑐_1_ × rand () × (Pbest*_i_* − 𝑥*_i_*) + 𝑐_2_ × rand () × (Gbest*_i_* − 𝑥*_i_*) (2)

where 𝑐_1_ and 𝑐_2_ are acceleration coefficients, predefined inertia is *w* and rand () is a random number function that generates any arbitrary number in the range [0, 1]. 𝑐_1_ is connected to the best solution of each particle, while 𝑐_2_ is associated with the best solution of all the localities. The flowchart for the basic PSO algorithm is shown in Figure 6.

In the swarm initialization step, the swarm population size is defined, as is the dimensionality of the search space; random positions and velocities are assigned to each particle. During the evaluation, the algorithm determines the fitness (in this case, the position with regard to the objective function) of each particle. The update of the personal best (cognitive component) starts from the best position attained so far for that concrete particle (personal best), and if its current position has achieved a fitness that exceeds the personal best, its data are updated with the newly attained best position in the search space. The global best (social component) update is based on the information shared by each particle with its locally surrounding neighboring particles. The update is completed by the best position in search space attained by any particle in the local neighborhood. Velocity is updated based on the inertia component *w*, the personal best of the particle and the global best. In this way, the trade-off between exploitation and exploration is controlled. The desired end is reached if the targeted fitness value is attained.

However, PSO can become trapped in a local optimum because of specific constraints in the exploration phenomena, especially when functions have multiple local optima. Over the years, numerous PSO modifications and upgrades have been put forth by researchers as solutions to this problem. Among them are PSO with time-varying acceleration coefficients [129], in which the rates of social and cognitive learning varied over time; human behavior-based PSO [130], which imitates human behavior by incorporating negative traits in humans by using the term “Gworst”; and PSO with aging leaders and challengers (ALCPSO) [131], where a leader is initially assigned, and as the leader ages, a new particle challenges its dominance. When coping with unimodal problems, these algorithms work well, but as the algorithm is moved toward more intricate and multimodal functions, the performance starts to deteriorate.

#### 4.2.2. Ant Colony Optimization (ACO)

ACO is the second most popular swarm intelligence algorithm, with more than 16,000 publications as of May 2023 [97]. This bio-inspired metaheuristic algorithm is based on the foraging behavior of ants in ant colonies.

It is known that ants as a collective find the shortest paths between their nest and the food sources due to worker ants leaving pheromone trails behind themselves and the rest of the legion of workers simply following these trails.

Each ant (*k*) in the ant colony mimicked by ACO will arbitrarily select a route, creating a graph structure and generating pheromones at the edges of the graph as it does so. The probability of choosing the route is calculated as

(3) $$P_{ij}^{k}=\left\{\begin{array}{c} \frac{{\left(\tau_{ij}\right)}^{\alpha }{\left(\eta_{ij}\right)}^{\beta }}{\sum_{l\epsilon J_{i}^{k}}{\left(\tau_{ij}\right)}^{\alpha }{\left(\eta_{ij}\right)}^{\beta }}\;\;\;\;\;\;\;\;\;\;\;\;\;\;\;\;\;\;\;\;j\notin \;J_{i}^{k}\;\;\;\;\;\;\;\;\;\\ \;0\;\;\;\;\;\;\;\;\;\;\;\;\;\;\;\;\;\;\;\;\;\;\;\;\;\;\;j\notin \;J_{i}^{k} \end{array}\right\}$$

Here, $J_{i}^{k}$ is the set of neighbors of vertex *i* of the *k^th^* ant, $\tau_{ij}$ represents the amount of pheromone trace on the edge (*i, j*), *α* and *β* are the weight factors that influence the pheromone trail and $\eta_{ij}$ is the visibility value. In contrast to the other paths, where the pheromone evaporation rate is such that pheromones are partially evaporated, the route providing the minimal objective function has its evaporation rate spike in each iteration (4).

(4) $$P_{ij}^{k}=\left\{\left(1-\rho \right)\tau_{ij}+\overset{m}{\underset{k=1}{\sum }}\Delta {\tau_{ij}}^{k}\right\}$$

where *m* is the number of ants, *ρ* is the pheromone evaporation rate and $\Delta {\tau_{ij}}^{k}$ is the quantity of pheromone laid on the edge (*i*, *j*) by ant *k*.

**Figure 7:**
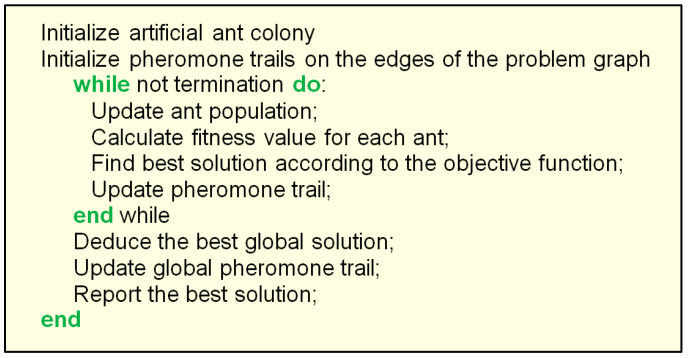
Pseudocode of ACO algorithm.

**Figure 8:**
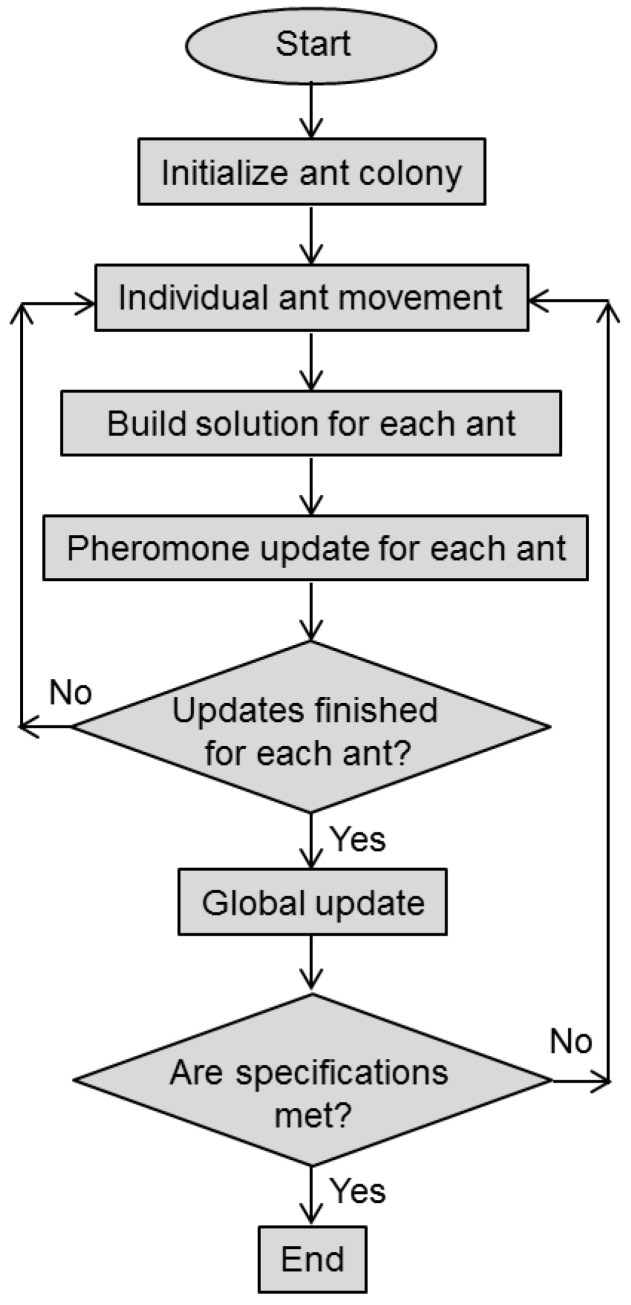
Flowchart of ACO algorithm.

The pseudocode of the ACO procedure is shown in Figure 7. The flowchart of the ACO is presented in Figure 8.

As shown in the flowchart, during the initialization step, a colony of ants is generated, where each individual ant represents a potential solution to the optimization problem and the initial pheromone levels are defined. During the individual ant movement step, each ant moves from one position (node) to another. The target node is chosen by its attractiveness, which is heuristically determined. The solution is built for each individual ant by selecting the node and by the ant moving along the node edges based on the pheromone level and the attractiveness of the node (again heuristically determined). A pheromone update to strengthen the trails is performed after every ant reaches its individual solution, the pheromone amount being proportional to the fitness of the individual solution. The global update is related to the pheromone levels, and it is performed according to the best solution found until that moment. The procedures are iteratively repeated until the desired solution is attained or the maximum number of iterations is exceeded.

#### 4.2.3. Whale Optimization Algorithm (WOA)

This swarm intelligence algorithm is based on the social behavior of humpback whales. More concretely, it mimics their peculiar method of hunting fish schools for food. These highly intelligent creatures developed their own hunting strategy: after their leader (alpha) encounters the target fish school, it starts circling around it, at the same time blowing bubbles that create a kind of net around the fish, preventing them from escaping it. The circling is a spiral and closes in on the prey. Another whale, supporting the leader, emits a call for the others to make a formation behind the leader, assume their attack positions and prepare to lunge at the prey. This rather complex maneuver is called bubble-net hunting.

A simplified procedure mimicking the bubble-net foraging attacks used by humpback whales when they are hunting their prey is used in the WOA optimization algorithm. The algorithm was proposed by Mirjalili and Lewis in their highly cited paper from 2016 [105]. This algorithm is population-based. It consists of three stages—exploration, exploitation and convergence (spiraling—local search).

The algorithm proceeds as follows: At the beginning, a population of “whales” is initialized in a random manner. Each individual whale represents a potential solution to the optimization problem, The whale is regarded as a particle; i.e., it is described solely by its position in the search space. In the exploration stage, the whales modify their position according to their current location and the best solution found until that stage. The aim of exploration is to diversify the search by the whales approaching the promising regions of the search space. Their position change is described by the following equations [105]:

During encircling the prey,

(5) $$\vec{D}=\left|\vec{C}.{\vec{X}}_{best}(t)-\vec{X}(t)\right|.$$

Updating the position of the current whale towards the best solution by encircling is accomplished as

(6) $$\vec{X}(t+1)={\vec{X}}_{best}(t)-\vec{A}\;\vec{D},$$

where $\vec{D}$ is the distance vector, *t* is the current iteration number, $\vec{A}\;$ is a coefficient and point (.) denotes element-by-element multiplication; $\vec{X}$ represents the position vector.

For spiral position updating, the following is valid:

(7) $$\vec{X}(t+1)=\vec{D}\prime \;\exp(bl)\;\;\cos(2\pi l)+{\vec{X}}_{prey}(t),$$

where $\vec{D}=\left|\vec{C}\;{\vec{X}}_{best}(t)-\vec{X}(t)\right|$ is the distance between the *i*th whale and the targeted prey and represents the best solution encountered until that moment, *b* is a constant parameter that defines the spiral shape and *l* is a random number in the [−1, 1] interval.

(8) $$\vec{X}(t+1)={\vec{X}}_{rand}-\vec{A}\;\vec{D}.$$

If *p* denotes the probability of whales choosing encircling or spiraling, then Equation (6) is valid for, e.g., *p* > 0.5, and Equation (7) is valid for *p* < 0.5.

**Figure 9:**
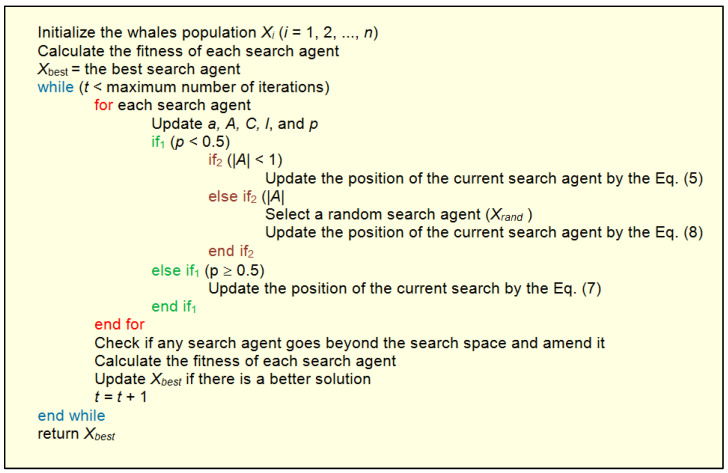
Pseudocode for the basic whale optimization algorithm.

As the algorithm progresses, the whales converge towards the global optimum. The convergence is achieved by gradually reducing the search space according to the presented procedure. For the whale optimization procedure in its basic form, a pseudocode may be written as shown in Figure 9.

**Figure 10:**
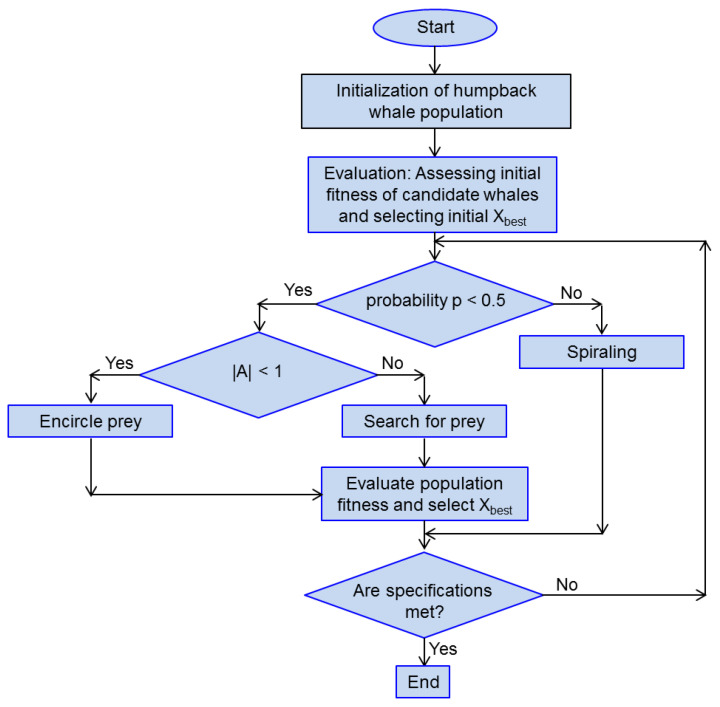
Whale optimization algorithm flowchart.

A possible flowchart for the whale optimization algorithm is shown in Figure 10. Numerous different versions of this algorithm exist, some of them generally improved, some of them tuned for a particular application. Hybrid and multi-objective versions are also encountered.

#### 4.2.4. Grey Wolf Optimizer (GWO)

The GWO algorithm is a swarm-intelligence-based metaheuristic procedure inspired by the social behavior and hunting strategies of wolfs in packs. It is assumed that in dependence on their roles in their packs, grey wolves are socially organized as a pyramidal hierarchy, although that may not be so in reality in the wild, and some prestigious sources have even described it as a myth [132].

Realistic or not, the often-described scheme is the source of the popular and very useful optimization grey wolf optimizer algorithm that was proposed in 2014 by Mirjalili, Mirjalili and Lewis. The metaheuristic optimizer can be presented as follows: At the top of the pyramid is Alpha, and he is the leader of the pack and the decision maker. Beta is the next in order; he helps Alpha in making decisions and disciplines the pack. He is also the candidate for the next Alpha. Delta is an average wolf, a “soldier” following Alpha and Beta in the hunt. Omega is the weakest in the pack and lowest in ranking.

According to the ranks of the grey wolves that execute the hunting process, the GWO algorithm is also organized into four groups. These hunting categories are also called alpha, beta, delta and omega, with alpha here denoting the most successful search strategy.

Similar to the previously described SI-based algorithms, the GWO search begins by establishing a random population of grey wolves. The four wolf groups are then formed, the positions of individuals are determined, and the distances to the intended prey are calculated. During the search process, each wolf is a particle that symbolizes a potential solution and is updated. In order to maintain exploration and exploitation and prevent the local optimum from stagnating, GWO additionally uses operations that are controlled by two factors. GWO just needs one vector of position; hence, it uses less memory than the PSO algorithm [21]. Additionally, while PSO preserves the best solution so far obtained by all particles as well as the single best solution for every particle, GWO only retains the three best solutions.

The standard GWO algorithm is initialized by setting the number of pack members to *n*, the parameter *a* that gradually decreases its value from 2 to 0, the maximum number of iterations *t_max_*, and the search agents ${\vec{X}}_{i}$, where *i* = 1, 2, …, *n* for a fitness function $f({\vec{X}}_{i})$. The three best solutions are, according to the pack hierarchy, denoted as ${\vec{X}}_{\alpha },\;\;{\vec{X}}_{\beta },\;{\vec{X}}_{\gamma }$. The distances to the target are described as

(9) $$\begin{array}{l} {\vec{D}}_{\alpha }=\left|{\vec{C}}_{1}{\vec{X}}_{\alpha }-\vec{X}\right|\\ {\vec{D}}_{\beta }=\left|{\vec{C}}_{2}{\vec{X}}_{\beta }-\vec{X}\right|\\ {\vec{D}}_{\delta }=\left|{\vec{C}}_{3}{\vec{X}}_{\delta }-\vec{X}\right| \end{array},$$

and the search agents (solutions) are

(10) $$\begin{array}{l} {\vec{X}}_{1}=\left|{\vec{X}}_{\alpha }-{\vec{A}}_{1}{\vec{D}}_{\alpha }\right|\\ {\vec{X}}_{2}=\left|{\vec{X}}_{\beta }-{\vec{A}}_{2}{\vec{D}}_{\beta }\right|\\ {\vec{X}}_{3}=\left|{\vec{X}}_{\delta }-{\vec{A}}_{3}{\vec{D}}_{\delta }\right| \end{array}.$$

The next iteration step is calculated as

(11) $$\vec{X}(t+1)=\frac{{\vec{X}}_{1}+{\vec{X}}_{2}+{\vec{X}}_{3}}{3},$$

where $\vec{A}=2\vec{a}$·${\vec{r}}_{1}$·$\vec{a}$ and $\vec{C}=2{\vec{r}}_{2}$ are coefficients, while ${\vec{r}}_{1},\;{\vec{r}}_{2}$ are random vectors with intensities in the range from 0 to 1.

**Figure 11:**
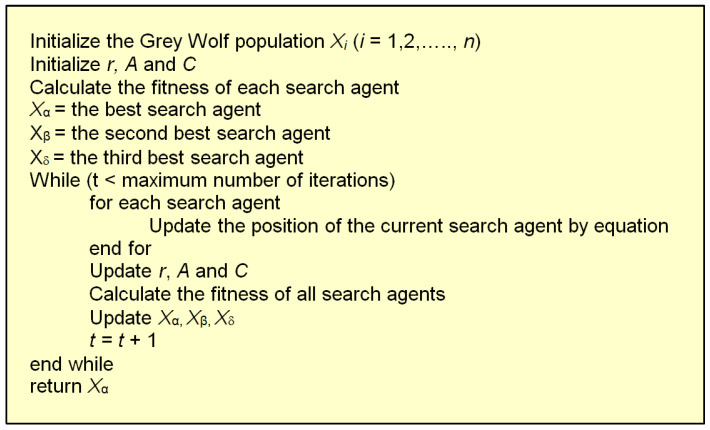
Pseudocode of the grey wolf optimizer.

**Figure 12:**
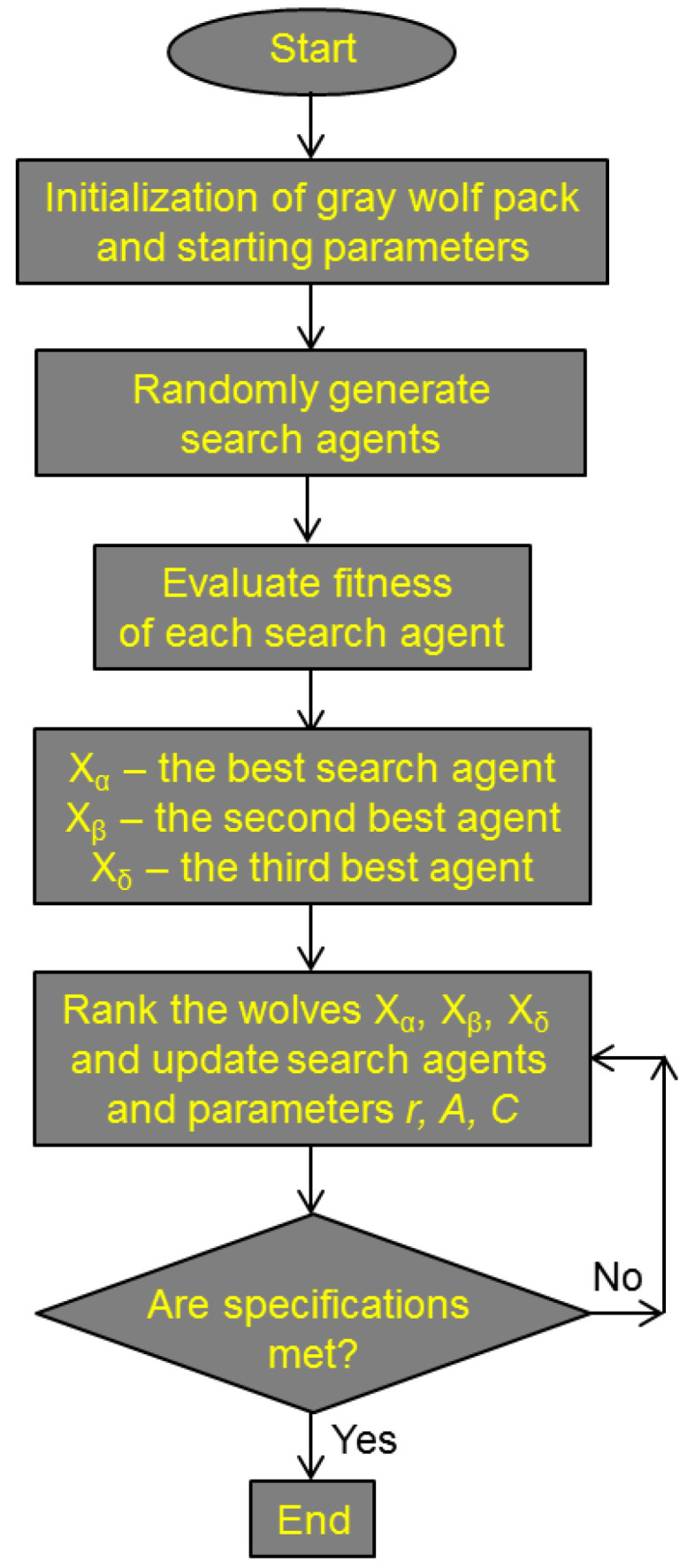
Flowchart of the grey wolf optimizer.

The pseudocode of the grey wolf optimizer is given in Figure 11. The flowchart of the GWO is shown in Figure 12.

#### 4.2.5. Firefly Optimization Algorithm (FOA)

Fireflies emit bioluminescent light at night to communicate among themselves and to attract mates. The attraction is based on the light intensity of an individual firefly (the stronger it is, the stronger the allure) and on its position (the apparent brightness of nearer fireflies is stronger).

A metaphor-based algorithm inspired by the attraction between fireflies was proposed by Xin-She Yang in 2008 [110]. The main expression driving the movement of the fireflies is

(12) $$x_{i}^{t+1}=x_{i}^{t}+\beta_{0}e^{-\gamma r_{ij}^{2}}\left(x_{j}^{t}-x_{i}^{t}\right)+\alpha \varepsilon_{i}^{t},$$

where $x_{i}^{t+1}$ is the updated position of an individual firefly (iteration step *t* + 1), $x_{i}^{t}$ is the current position (iteration step *t*), $\beta_{0}$ is the attractiveness between a pair of fireflies at zero distance and γ denotes the light absorption coefficient (the bracketed term multiplied by $\beta_{0}$ represents a light intensity decrease due to distance and the light absorption of the atmosphere). In the last term on the right, α denotes a scaling factor, and $\varepsilon_{i}^{t}$ is a random vector defining perturbation.

The algorithm in its basic form works as follows: First, a population of fireflies, each representing a potential solution, is randomly initialized. They are considered as particles, meaning that their position in search space actually defines a candidate solution. Their movement is based on the attraction to other fireflies, which is larger if the bioluminescent glow of a neighboring firefly is stronger or the neighboring firefly is nearer. The brightness is defined by the objective function related to the individual’s position in the search space. The firefly positions are updated according to Equation (12) which includes both the distance and the real intensity of the glow. The firefly positions are iteratively updated. In this way, the promising regions of the search space are exploited.

**Figure 13:**
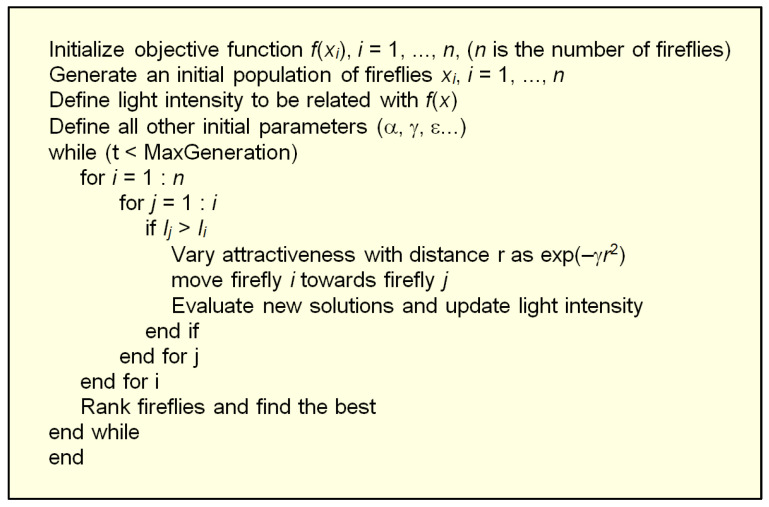
Pseudocode for firefly optimization algorithm.

**Figure 14:**
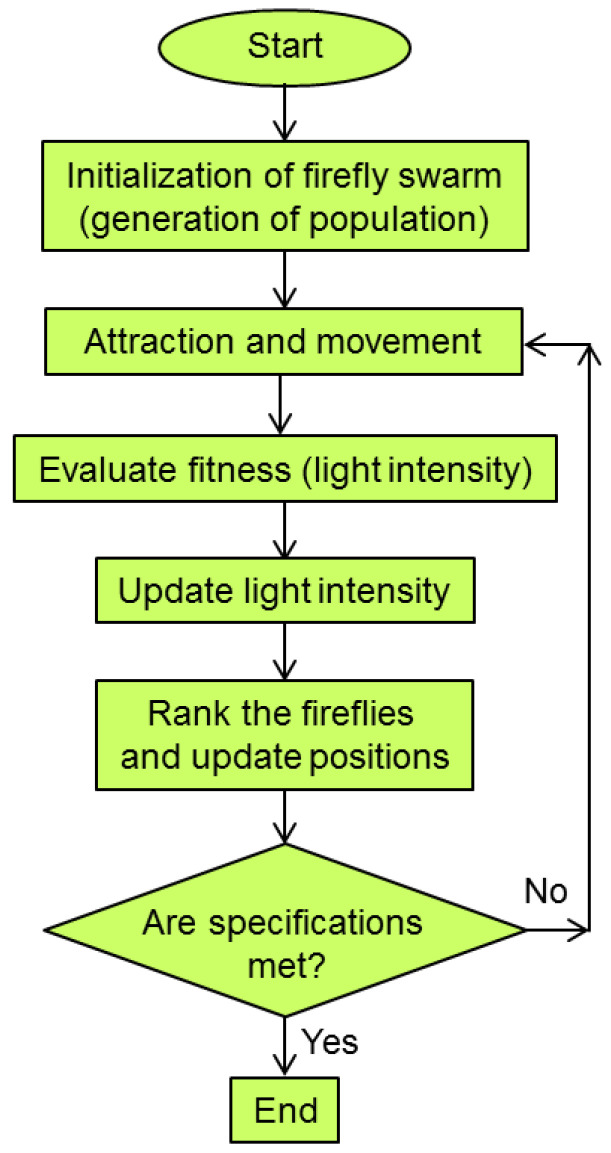
A flowchart of the algorithm.

A pseudocode for the basic firefly optimization algorithm is given in Figure 13. A flowchart of the algorithm is presented in Figure 14.

#### 4.2.6. Bat Optimization Algorithm (BOA)

The BOA is a global metaheuristic algorithm that simulates the echolocation of microbats belonging to the zoological suborder Microchiroptera. To locate their prey, microbats emit sound pulses typically in the range from 14 kHz to 200 kHz, i.e., for the most part far from the human hearing range, constantly varying the pulse frequency, loudness and pulse rate. The echo reflected from their prey enables them to locate, approach and catch it. The bat optimization algorithm is based on that behavior. It was proposed by Xin-She Yang in 2010 [133].

Despite its simple design, the BOA has proven itself to be effective. The bat optimization algorithm can sometimes result in an imbalance between exploration and exploitation in order to find the true global solution if the parameters utilized are not adjusted appropriately. As a result, numerous studies have developed a variety of hybridized and modified bat algorithms to boost their efficiency and find overall solutions to optimization problems [134]. Other varieties of BOA have been proposed as enhancements and adaptations to many practical situations.

The optimization proceeds in the following manner: At an iteration number *t*, each individual bat is allotted a velocity $v_{i}^{t}$ and a location $x_{i}^{t}$ in a multidimensional search space. Here, *i* denotes the number of an individual microbat. There exists a specific best answer, *x_best_*, amongst all the bats. Equations (13)–(15) help in updating the positions and velocities:

(13) $$f_{i}=f_{min}+\left(f_{max}-f_{min}\right)\beta ,$$

(14) $$v_{i}^{t}=v_{i}^{t-1}+\left(x_{i}^{t-1}-x^{*}\right)f_{i},$$

(15) $$x_{i}^{t}=x_{i}^{t-1}+v_{i}^{t},$$

where *β* ∈ [0, 1] is an arbitrary vector obtained from a uniform distribution. Each bat is initially given a frequency *f* that is randomly picked from the range [*f_min_, f_max_*]. Due to this, the bat algorithm can be viewed as a frequency-tuning algorithm that offers a balanced combination of exploitation and exploration [135].

A system for automatic control and auto-zooming into the area with potential options is fundamentally provided by the loudness and pulse emission rates. BOA uses a simple monotonic form for both loudness *A* and pulse emission rate *r*, although in reality, these may have quite complex forms. They are defined as

(16) $$A_{i}^{t+1}=\propto A_{i}^{t},$$

(17) $$r_{i}^{t+1}=r_{i}^{t}\left(1-e^{-\gamma t}\right).$$

**Figure 15:**
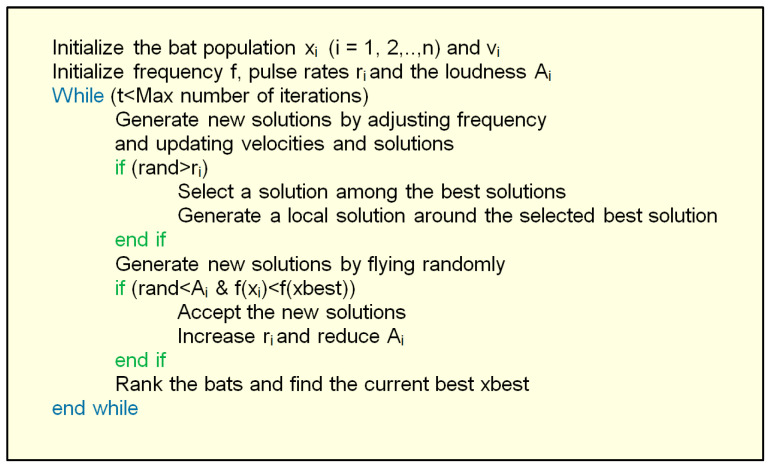
Pseudocode of the bat optimization algorithm.

**Figure 16:**
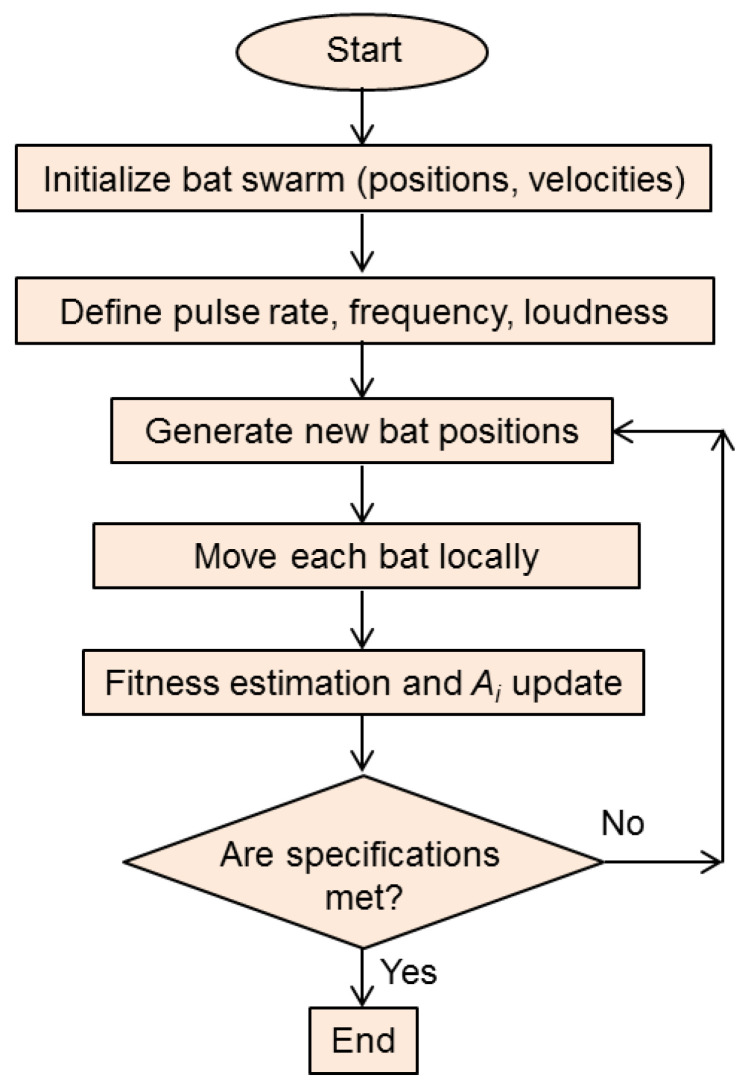
Flowchart of the bat optimization algorithm.

The pseudocode of the BOA procedure is shown in Figure 15. It clearly shows the iterative nature of the optimization process. Figure 16 shows the flowchart of the BOA.

#### 4.2.7. Orca Predation Algorithm (OPA)

The orca predation algorithm (OPA) is one of the more recent swarm intelligence algorithms, being proposed in 2022 [123]. It should not be confused with some other similarly named metaheuristics, including killer whale algorithm [136], ORCA optimization algorithm (OOA) [137], orcas algorithm (OA) [138] and artificial orcas algorithm (AOA) [139] (which all have a lower citation count per year, and some of them even have no citations at all), or with some other programming systems totally unrelated to either optimization algorithms or biomimetics. The OPA mimics the hunting behavior of killer whales (Orcinus orca), creatures whose intelligence is comparable to that of humans. When they hunt their prey, they do so in packs, using echolocation to find it and communicating among themselves using their sonars to exchange information and coordinate their attacks. Instead of being based on a random feeding frenzy, their attacks are highly coordinated and planned. This makes them formidable predators; actually, orcas are apex predators of the ocean, preying even on sharks. The ruthless efficiency of their hunting skills motivated the creation of the OPA.

The algorithm divides the procedure into three mathematical sub-models: (1) searching for prey, (2) driving and encircling it and (3) attacking it. In order to introduce a balance between the exploration and exploitation stages, different weight coefficients are assigned to different stages of prey driving and encircling, and the algorithm parameters are adjusted to achieve that goal. More concretely, the positions of those killer whales which are superior are ascertained, as are the positions of those which are average and randomly chosen. In this way, the optimal solutions are approached during the attack stage, while at the same time, the diversity of individual killer whales is fully retained.

Different stages of the OPA can be described by the steps corresponding to the real-world hunt of an orca pack. The first step is the establishment of the pack itself. It is considered to be a population of *N* particles in the search space (potential solutions), and the search space has *D* dimensions, so the orca pack population can be described as $X=[x_{1},\;\;x_{2,}\;\ldots \;x_{N}]$. Here, $x_{N}$ denotes the position of the *N*th individual orca in the pack. The second step is searching for the prey, typically a school of fish. After one of the orcas has spotted a school, the chasing phase begins. The orca pack disperses and starts the first stage of the chase—the driving, in which the whole school is driven by orcas towards the water’s surface. The driving can proceed according to one of two different procedures, in dependence on the size of the orca pack. The pack may be small (case 1) or large (case 2). To describe this situation, a probability *q* from the interval [0, 1] is used. The algorithm generates a random number rand, and if the orca pack is large, then rand > *q*, and the first method of driving will be used; if the orca pack is small (rand ≤ *q*), the second method will be used. The two methods are described by the equations

(18) $$V_{chase,1,i}^{t}=a\;\left(d\;x_{best}^{t}-2\;(b\;M^{t}+c\;x_{i}^{t}\right),$$

(19) $$V_{chase,2,i}^{t}=e\;\;\;x_{best}^{t}-\;x_{i}^{t}\;,$$

where *t* is the number of the current iteration; *V_chase_* is the chasing speed of orcas; *a*, *b* and *d* are random coefficients from the [0, 1] range, and *e* is a random coefficient from the range [0, 2]; the numbers 1 and 2 in the subscripts denote the first (18) or the second (19) mentioned driving strategy; *x* is the position of *i*th orca particle; the subscript “best” denotes the best solution for *x*; *i* is the number of the orca under consideration; and *M* is the average location of the orca pack (solution population) defined as

(20) $$M^{t}=\frac{\sum_{\;\;\;i=1}^{\;\;\;N}x_{i}^{t}}{N}\;.$$

The positions of orca particles during the driving procedure will be determined by

(21) $$x_{chase,1,i}^{t}=x_{i}^{t}+V_{chase,1,i}^{t}\;\;\;\;\;\;\;\;\mathrm{rand}\;>\;q,$$

(22) $$x_{chase,2,i}^{t}=x_{i}^{t}+V_{chase,2,i}^{t}\;\;\;\;\;\;\;\;\mathrm{rand}\;\leq \;q.$$

After the driving has ended, the encircling stage begins. In that way, orcas force the fish from the school to form a roughly spherical and tightly packed formation. If three orcas are randomly selected, other orcas will follow them during encircling, and the position of the *i*th orca will be determined according to their position as

(23) $$x_{chase,3,i,k}^{t}=x_{d1,k}^{t}+2\;(rand-\;\frac{1}{2})\frac{t_{\max}-t}{t_{\max}}\;(x_{d2,k}^{t}-x_{d2,k}^{t})\;.$$

The orca positions during encirclement are changing according to

(24) $$x_{chase,i}^{t}=x_{chase,i}^{t}\;\;\;\;\;\;f(x_{chase,i}^{t})<f(x_{i}^{t})\;,$$

(25) $$x_{chase,i}^{t}=x_{i}^{t}\;\;\;\;\;\;\;\;\;\;\;\;\;\;\;f(x_{chase,i}^{t})\geq f(x_{i}^{t})\;,$$

where *f* is the objective function.

In the final, attacking stage, the algorithm chooses the four best-positioned orcas to attack. Their positions and speeds are calculated as

(26) $$V_{attack,1,i}^{t}=(x_{1}^{t}+x_{2}^{t}+x_{3}^{t}+x_{4}^{t})/4-x_{chase.i}^{t},$$

(27) $$V_{attack,2,i}^{t}=(x_{chase,d1}^{t}+x_{chase,d2}^{t}+x_{chase,d3}^{t})/3-x_{i}^{t},$$

(28) $$x_{attack,i}^{t}=x_{chase,i}^{t}+g_{1}\;V_{attack,1,i}^{t}+g_{2}\;V_{attack,2,i}^{t},$$

where *V* denotes speed; numbers 1 to 4 denote orcas in the best positions; *d*_1_, *d*_2_ and *d*_3_ are randomly chosen orcas from the pack of *N*; and the subscript attack denotes that the value is valid for the attacking stage. The parameter *g*_1_ is a randomly generated number in the range [0, 2], while *g*_2_ is a randomly generated number in the range [−2.5, 2.5].

**Figure 17:**
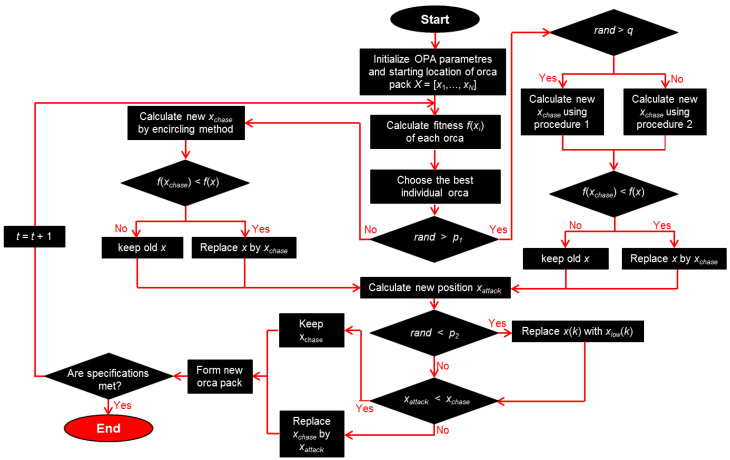
Flowchart of the orca predation algorithm in its basic form.

The flowchart of the orca predation algorithm is shown in Figure 17. It is based on the diagram from [123], but with some modifications. The parameter *x_low_* represents the lower boundary of the problem. Although complicated at first look, the algorithm is rather simple in the mathematical sense, so its calculation speed is comparatively high [123].

#### 4.2.8. Starling Murmuration Optimizer (SMO)

**Figure 18:**
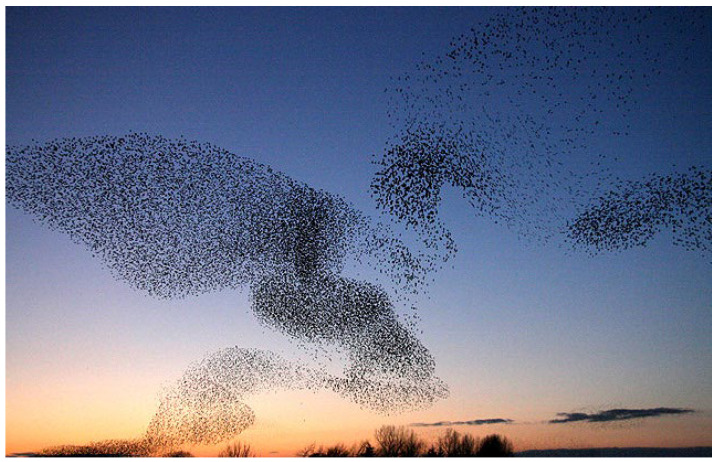
Starlings over Gretna by Walter Baxter, 29 November 2008, © Copyright Walter Baxter under Creative Commons Attribution-Share Alike 2.0 Generic (CC BY-SA 2.0), image retrieved from https://commons.wikimedia.org/wiki/File:Starlings_over_Gretna_-_geograph.org.uk_-_1069349.jpg, (accessed on 2 June 2023). No changes were made to the original.

Starling murmuration is a magnificent display of the collective behavior of starlings (family Sturnidae) when huge flocks of countless birds swoop and swirl in intricately synchronized shape-shifting living clouds, creating amazing visual spectacles [140] (see Figure 18). The motive for the use of that behavior in optimization is that not a single bird among the countless thousands of them flying in coordination ever collides with any other. It is no wonder that among the first practical applications of the SMO algorithm was the flight coordination of massive swarms of drones [141].

The starling murmuration optimizer (SMO) is a metaheuristic algorithm introduced in 2022 by Zamani et al. [124]. It is a population-based algorithm utilizing a dynamic multi-flock construction. It introduces three new search strategies: separating, diving and whirling.

At the initial step of the algorithm, individual starlings are stochastically distributed. The initial position of the *i*th starling in the group of *N* birds is described by

(29) $$x_{id}=x_{d}^{L}+rand(0,1)\;\left(x_{d}^{U}-x_{d}^{L}\right),\;\;\;i=1,2,\ldots N;\;\;\;\;d=1,2,\ldots D,$$

where *N* is the total number of starlings, *D* is the number of dimensions of the search space, $x_{id}$ is the *d*th dimension of the starling *s_i_*, $\;x_{d}^{U}$ is the upper bound of the search space and $x_{d}^{L}$ is the lower bound of the search space. *rand*(0, 1) is a random function with a value in the interval between 0 and 1.

After initialization, some of the starlings separate from the flock to form a new flock *P_sep_* that will explore the search space according to

(30) $$P_{sep}=\frac{\log(t+D)}{2\;\log(t_{\max})},$$

where *t* is the number of the current iteration and *t_max_* is the maximum number of allowed iterations. The search strategy is defined by

(31) $$X_{i}\left(t+1\right)=X_{G}(t)+\Xi_{1}(y)\;\;\left[X_{r\prime }(u)-X_{r}(t)\right],$$

where *X_G_*(*t*) is the global position obtained during the iterative step *t*, *X_r’_*(*t*) is the position selected from a proportion of the fittest starlings and the separated flock, and *X_r_*(*t*) is randomly selected from a population. The separation operator $\Xi_{1}(y)$ is a new operator based on the standard quantum harmonic oscillator, in which *y* represents a random number from the Gaussian distribution [124].

The starlings that remained after the separation dynamically construct the multi-flock with members *f*_1_, *f*_2_, …, *f_k_*. The quality *Q_q_*(*t*) of the *q*th flock is calculated by

(32) $$Q_{q}(t)=\frac{\sum_{\;i=1}^{\;\;k}\sum_{\;j=1}^{\;n}sf_{ij}(t)}{\frac{1}{n}\sum_{\;i=1}^{\;n}sf_{qi}(t)}$$

to select either the diving (exploration) or the whirling (exploitation) search strategy. The diving strategy explores the search space using a new quantum random dive operator, and the whirling strategy exploits the neighborhood of promising regions using a new cohesion force operator [124]. Here, *sf_ij_*(*t*) is the fitness value of the *i*th starling from the *j*th flock *f_j_*; *k* is the number of flocks in a murmuration *M*. The average quality of all flocks is denoted as μ*_q_*.

**Figure 19:**
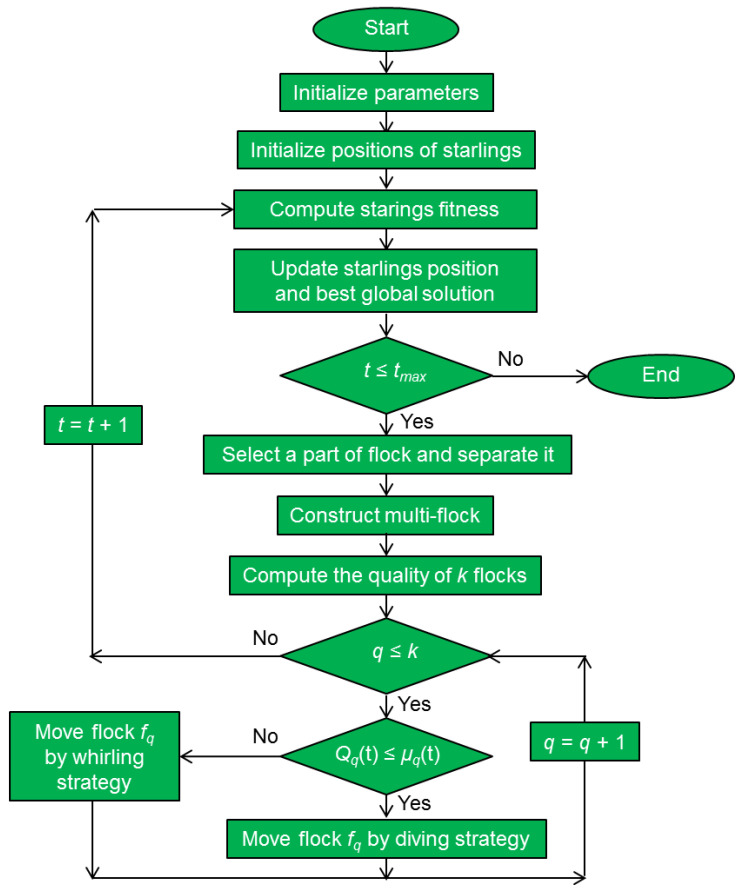
Flowchart of starling murmuration algorithm. Modified from [124].

Figure 19 shows the flowchart of the starling murmuration algorithm. Mathematically, the starling murmuration algorithm is rather complex because of the use of the new quantum operators.

### 4.3. Metaheuristics Mimicking Human or Zoological Physiological Functions

Physiological functions of humans or certain mammals have also served as bio-inspiration for some metaheuristic optimization methods. Table 4 shows just three such algorithms, and one of the most important and most often used ones among them, the artificial immune system (AIS), is presented in more detail in the subdivision that follows.

#### Artificial Immune Systems (AISs)

Artificial immune systems (AISs) are a class of algorithms mimicking the function of the human and generally vertebrate immune system. They are among the most popular optimization algorithms, with the number of published papers about AISs being about 22,000 as of May 2023 [97]. In dependence on how they are used, they can be classified both as metaphor-based metaheuristic algorithms [145,146] and machine learning techniques [147]. They belong to metaheuristic algorithms because they perform optimization tasks, explore the search space and perform iterative improvements to find approximately optimal solutions [145,146]. On the other hand, they also belong to machine learning techniques because they involve rule-based learning from data and include adaptive mechanisms (utilizing feedback information) [147]. The fact that they are bio-inspired and metaphor-based is a trait that differentiates them from conventional machine learning procedures. It may be said that AISs inherently represent a combination of metaheuristic algorithms with machine learning; i.e., the two areas overlap in them.

The function of an AIS algorithm is based on various functions of the immune system. After encountering antigens (any agents that our immune system sees as foreign and tries to fight off), immune cells trigger an immune response and produce antibodies or activate themselves. In the AIS algorithms, the immune response corresponds to the evolution-based adaptation of antibodies to improve their fitness or affinity to antigens (e.g., through modification of the existing ones or generation of new ones by way of mutation or recombination).

Since they represent a group of procedures, AISs include different algorithms. The most widely used and popular ones among those are the clonal selection algorithm, the artificial immune network algorithms, the negative selection algorithm, the dendritic cell algorithm, the danger theory, the humoral immune response, the pattern recognition receptor model and the artificial immune recognition system. Some of the mentioned types of the AIS algorithm group themselves present groups rather than a single algorithm. In further text, we give a short description of some of the most well known AIS algorithms.

*Clonal selection algorithms (CSAs)* are inspired by an acquired immunity mechanism called clonal selection. According to the theory of clonal selection, T cells (lymphocytes that attack and destroy foreign agents) and B cells (lymphocytes that make antibodies) achieve improvements in their response to antigens (substances and other agents that trigger a response of the immune system because the system does not recognize them and tries to fight them off—bacteria, viruses, toxins, allergens, foreign particulate matter, etc.) through the process of affinity maturation in which T-cell-activated B cells produce antibodies with more and more increasing affinity for a particular antigen during an immune response to exposures to that antigen. CSA algorithms focus on a “survival of the fittest” Darwinian selection process applied to the immune cells. In that kind of time-maturation process, the selection corresponds to the affinity of antigen-antibody interactions, reproduction corresponds to the cell division and variation corresponds to the somatic hypermutation (a mechanism at the cell level by which the immune system adapts to the new hostile foreign elements such as pathogen microorganisms). Antibodies with improved affinities are selectively cloned and mutated. Thus, a population of diverse improved solutions is generated.

*Artificial immune network algorithms* are inspired by the interactions of the immune cells within the immune system. The algorithm creates a network graph structure to represent candidate solutions. Each graph node corresponds to a potential solution. The training algorithm generates or removes the interconnections between the nodes on the basis of affinity (which corresponds to the similarity in the search space). This leads to the network graph evolution and promotes cooperation and competition among the network graph nodes.

*The negative selection algorithm* is inspired by the negative selection mechanism where the immune system identifies and kills self-reacting cells, i.e., the T cells that anomalously target and attack the organism’s own cells, by the process of apoptosis—programmed cell death. The algorithm establishes exemplary pattern “detectors” of self-components which it trains on normal cells, evolving them to recognize non-self patterns, achieving a high detection rate for anomalous patterns, while minimizing false positives. A population of antigen patterns (non-self patterns) is generated in the process.

*The dendritic cell algorithm* is based on mimicking the functions of dendritic cells (a type of phagocytes and a type of antigen-presenting cells that activate immune response and orchestrate the behavior of T cells). In this algorithm, candidate solutions are represented as antigens, while the role of dendritic cells is to capture and disable antigens. The algorithm implements a feedback system through which dendritic cells adapt themselves in dependence on their interaction with antigens. This is a multi-scale process since it involves different levels, starting from the molecule networks within a single cell to the whole population of cells.

*An artificial immune recognition system* combines various immune system elements, including an artificial immune network, negative selection and clonal selection. In other words, it combines different immune aspect-based algorithms to arrive at an improved and robust optimization procedure.

**Figure 20:**
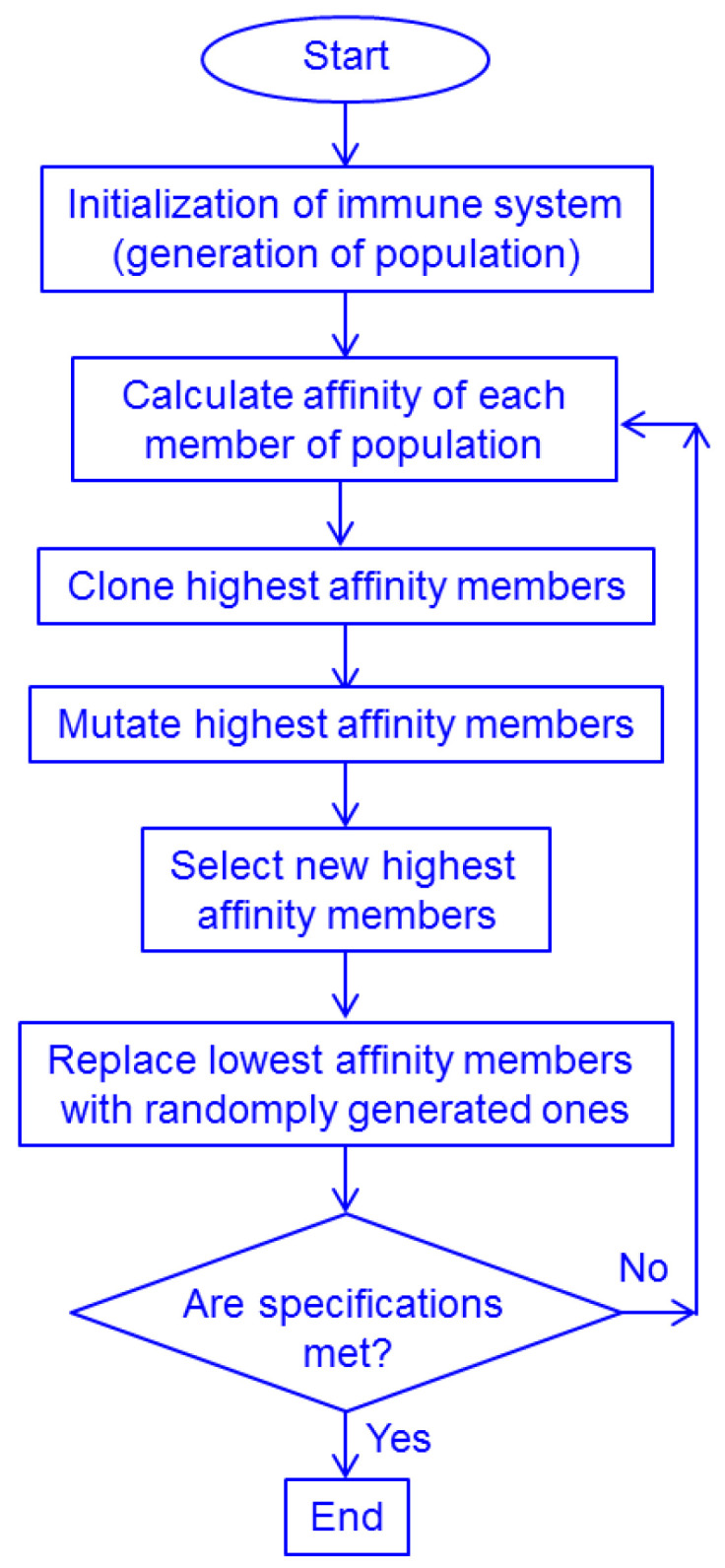
Flowchart of one of the clonal selection algorithms.

As an illustration of the way the AIS algorithms function, we present here the flowchart of one of the clonal selection algorithms. The flowchart is shown in Figure 20.

The particular properties of various types of AISs are often further modified and tailored since the algorithms from this group offer large and flexible customization possibilities. Over the years, different variants and extensions of the AISs have been introduced to adapt the algorithms to different specific applications and to improve their efficiency.

### 4.4. Anthropological Algorithms (Mimicking Human Social Behavior)

This subsection presents metaheuristic optimization algorithms inspired by human social behavior. The description of the contents of Table 2 is also valid for Table 5. In the single subdivision following Table 5, more details are given about the tabu search algorithm (TSA), although there is disagreement among different research teams regarding which metaheuristic group this algorithm actually belongs in. Our reasoning is that since tabu (or, spelled alternatively, taboo) is a social phenomenon present in practically all human societies and the algorithm draws its name from it, as well as its basic properties, it should merit inclusion in this group.

#### Tabu Search Algorithm (TSA)

As mentioned above, there is contradictory information in the literature on the correct classification of the tabu (taboo) search algorithm. Different researchers classified it in different metaheuristic groups, even among the mathematics-based nature-inspired algorithms [96]. However, since its main assumption is based on anthropological habits, it is more often than not categorized among human social behavior-based algorithms [158].

The word “tabu” (or, in an alternative and more frequently used spelling, “taboo”) is a Tongan expression for a sacred thing that is forbidden to be touched. Almost all human societies have their taboos in one form or another.

Tabu search is a single-solution-based metaheuristic optimization algorithm [97], contrary to all the other metaheuristics quoted here, which are population-based. It is designed to perform combinatorial optimization utilizing local search methodology. When searching for an improved potential solution, it checks the immediate neighboring solutions in the search space. A peculiarity of this algorithm is that it utilizes memory to remember the previously visited solutions and to prohibit their revisiting. In other words, these solutions become tabu. Solutions that are undesirable according to a user-defined rule or set of rules (the aspiration criteria) also become tabu. The algorithm makes a list of forbidden solutions and thus remembers them. Another peculiarity of the algorithm is that in situations when no improved solution exists (e.g., when stuck in a local minimum), the algorithm allows for choosing a worse solution—i.e., it relaxes its basic requirement of local search to always strive for a better solution. In this way, tabu search becomes a local search method that is able to escape local minima and continue searching for a global optimum.

**Figure 21:**
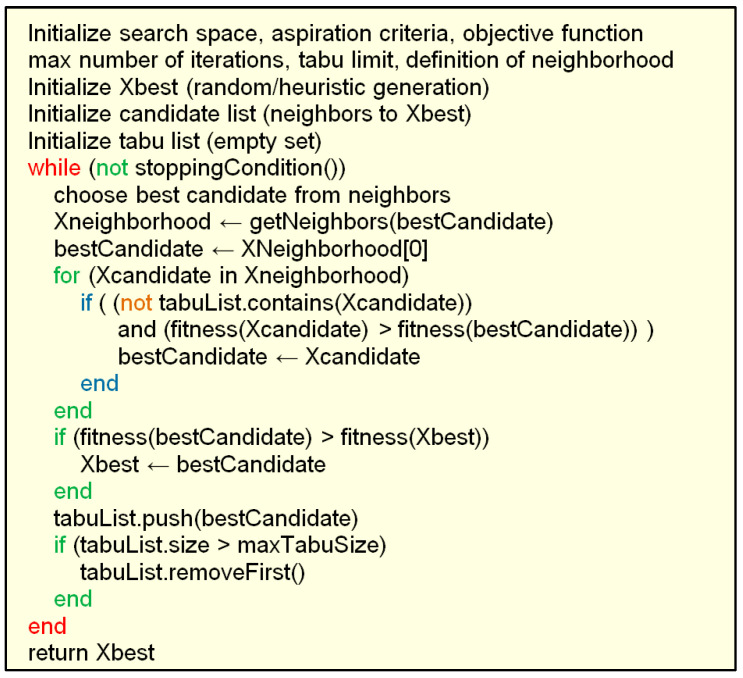
Pseudocode of the standard tabu search algorithm.

**Figure 22:**
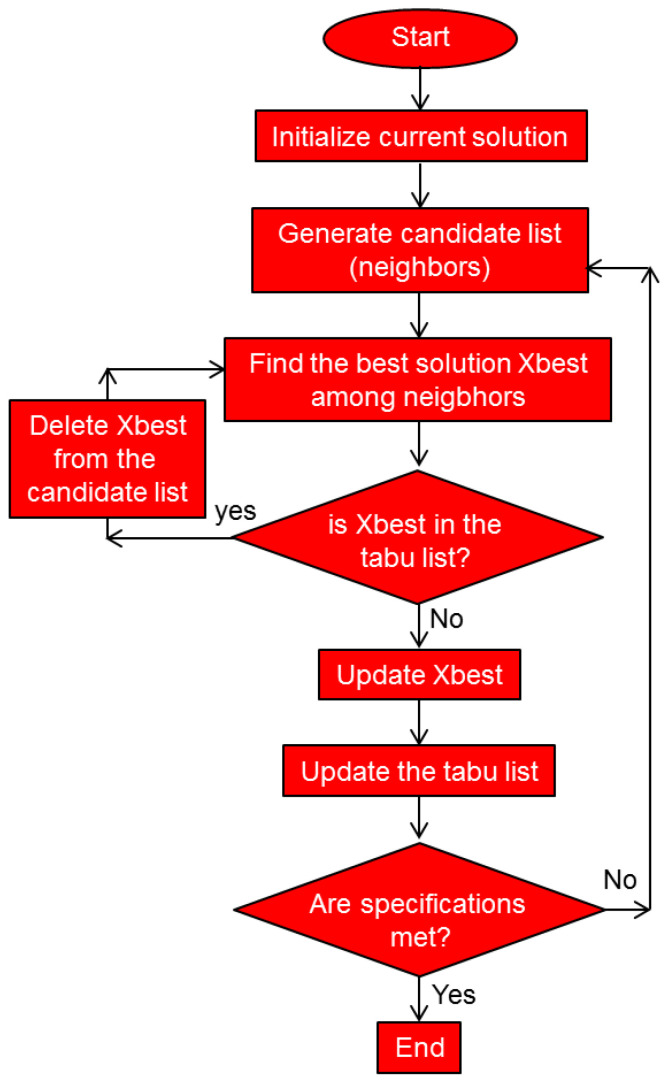
Flowchart of the standard tabu search algorithm.

Figure 21 shows the pseudocode of the standard tabu search algorithm. Figure 22 shows the flowchart of the standard tabu search algorithm.

### 4.5. Plant-Based Algorithms

This subsection presents metaheuristic optimization algorithms inspired by plant life properties. The Table 2 description is valid for the contents of Table 6. In the single subdivision following Table 6, the most popular plant-based algorithm, flower pollination algorithm (FPA), is described in more detail.

#### Flower Pollination Algorithm (FPA)

**Figure 23:**
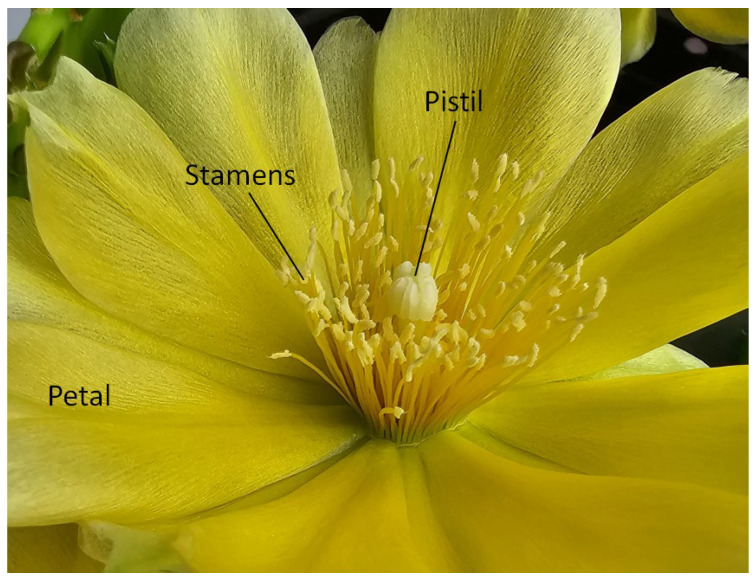
Stamens, petals and pistil of a flowering plant (*Opuntia ficus-indica*). Photo by Zoran Jakšić, 2023.

The clade of flowering plants (Angiospermae) represents the most advanced and most diverse land plants on the earth, with currently more than 300,000 known species. Among the reasons for such dominance in nature is their way of reproduction by pollination. They bear male and female reproductive cells. The male cells, the pollen, are borne in the stamens. The female cells are megaspores, and their division creates the egg cell. These are enclosed in the carpel, where one or more carpels form the pistil. As an example, Figure 23 shows the main reproductive parts of a flowering plant (a prickly pear flower).

Flowering plants may be pollinated (i.e., their pollen brought to the egg cell) in abiotic ways (by wind, rain or dew, or by sheer gravity) or biotic ways (i.e., by insects, birds or mammals). If a plant is pollinated in a biotic way, it probably will have developed a mechanism to attract its pollinator organisms. Most often, such plants form petals that may be brightly colored (sometimes petals are denoted as tepals, if the petals are indistinguishable from the protective sepals). Another way to attract biotic pollinators is through smell, which can be, in dependence on the desirable pollinator, fragrance from the human point of view or stench (e.g., resembling a rotten carcass, which attracts the targeted pollinators, e.g., blowflies). In abiotically pollinated plants, the petals and sepals may be completely absent (any appeal to animals being unnecessary). A flower may be self-pollinated (the pollen of a single flower pollinating the egg cell of the same flower) or cross-pollinated (two different flowers are needed for pollination). Such a system may appear complex, but it ensured the dominance and diversity of flowering plants.

The flower pollination algorithm (FPA) is a metaheuristic algorithm based on the pollination behavior of flowering plants. It was proposed by Xin-She Yang in 2012 in his widely cited conference paper [165]. The FPA is the most widely published and cited of all plant-based algorithms, having appeared in about 1000 dedicated publications as of May 2023 [97]. In the basic version of the algorithm, it is assumed that each plant bears a single flower, where each flower produces only one grain of pollen. In this way, the candidate solution is the flower or its grain of pollen. The motion through the search space is accomplished by biotic cross-pollination. The movement of each grain of pollen is represented by Lévy flight (a type of random walk), a method that has spread to other metaheuristics too [166]. The FPA allows exchange of information and the choice of improved solutions. In this way, it promotes the exchange of “knowledge” between different flowers/candidate solutions and thus enhances the exploration stage.

A typical FPA procedure begins by initializing a starting flower/pollen grain population, where the positions of the pollen grains represent the candidate solutions. Corresponding to the natural pollination process, FPA allows the flowers to interact and exchange information in a search for better solutions in several manners. A flower is selected for reproduction according to its fitness/objective function value and becomes the pollinating flower (the source). It then perturbs its position in the search space through a random mechanism (controlled by a randomization factor) and generates a new solution. It can be procreated by randomly choosing local or global pollination. The fitness of the solution (offspring) is compared to the pollinator flower. In dependence on their values of fitness, the original flower is replaced or retained. These steps are repeated for the whole flower population. Exploration is thus accomplished thorough random perturbations, while the exploitation proceeds through the selection process based on the fitness criterion. If a satisfactory solution is found, or a maximum number of iterations is reached, the algorithm terminates.

**Figure 24:**
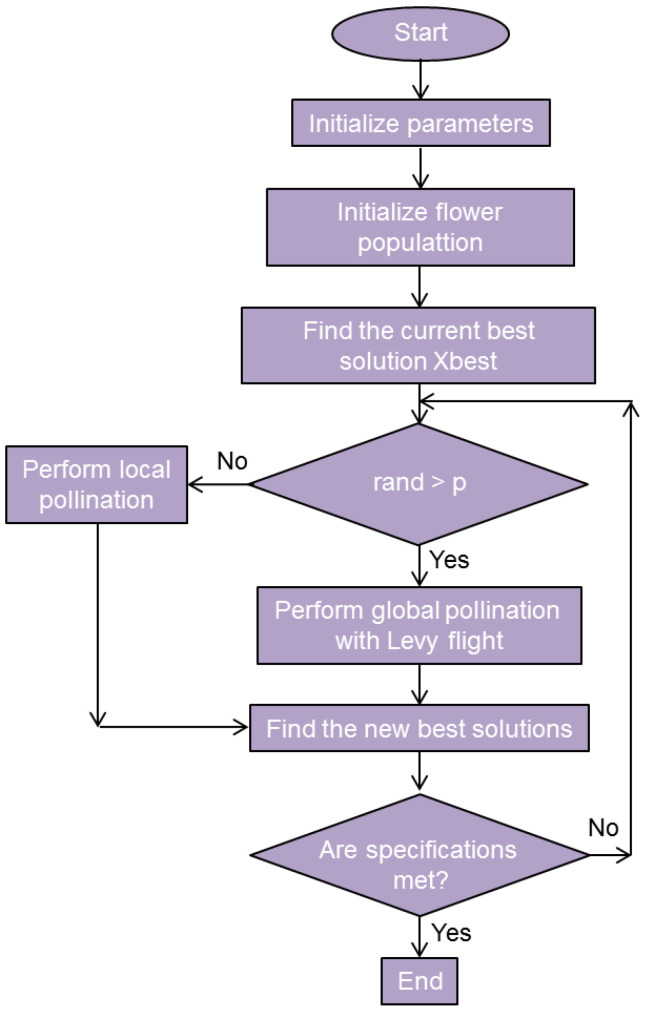
Flowchart of flower pollination algorithm.

A pseudocode for the FPA can be found in [159]. Equations describing the FPA, including the procedure for the calculation of the best solution and those for Lévy flight, can be found in the same article. Figure 24 presents a flowchart for the FPA.

There are numerous improved variations and upgraded alternative versions of the FPA. Among others, FPA procedures were written for multi-objective optimization [167]. Many hybrids of the FPA with other metaheuristics were reported, as well as with mathematical optimization methods and with machine learning methods [168].

## 5. Hyper-Heuristics

The hyper-heuristic algorithms [169] were first introduced as “heuristics to choose heuristics”, i.e., an approach to automate the selection or design (generation) of metaheuristic algorithms to be able to solve the most difficult optimization problems. The term was coined by Cawling, Kendall and Soubeiga in 2000 [170]. Hyper-heuristics may be a learning method or a search procedure. They use conventional heuristics or metaheuristics as their “base” and explore them, seeking strategies to combine them, select the most convenient ones among them or generate the optimal ones. Thus, a hyper-heuristic algorithm operates in the search space of heuristics/metaheuristics, in contrast to ordinary heuristics/metaheuristics which operate in the search space of an optimization problem. Its goal is to reach a generality instead of targeting a specific problem space. The goal of hyper-heuristic algorithms is to find effective strategies through a high-level approach that are adaptable to a range of different problems and problem domains. Regardless of the methodology used, one can implement them as iterative procedures, where a sequence of lower-level algorithms keeps reiterating, all the time attempting to improve the solution(s) from the previous step. Reinforcement learning techniques [171,172] can be also utilized to automatically learn and improve the hyper-heuristic procedure.

A possible workflow for hyper-heuristics includes the initialization step where a set of base heuristics or their constitutive parts is selected or generated, followed by an iterative exploration of the search space of possible heuristics/metaheuristics or their parts, adapting or refining the available heuristics or generating new ones. The final step is the performance evaluation of the obtained solutions in the meta-search space and, in dependence on its results, arrival at the termination criteria. A few common hyper-heuristic approaches are briefly presented below.

### 5.1. Selection Hyper-Heuristics

The selection of hyper-heuristic algorithms comprises a group of already existing and available heuristic/metaheuristic algorithms that evaluate their performance within the context of a current problem and select the most promising one among them according to given criteria. These criteria may be based on a previous experience with the application of pre-existing heuristics/metaheuristics on a similar type of optimization problem. Besides that kind of history-based approach, a set of criteria may be based on the features that are problem-specific. Besides the selection of a single existing algorithm that best fits the current problem, there is a possibility to learn connections between partial stages of solving a problem and the most convenient heuristics/metaheuristics for those stages.

The two main methodologies used for hyper-heuristic selection are (1) approaches utilizing constructive low-level heuristics/metaheuristics (incrementally and intelligently building a solution, starting from an empty set) and (2) approaches utilizing perturbative low-level heuristics/metaheuristics (utilizing automatic selection and applying heuristics to improve a candidate solution) [169].

### 5.2. Generation Hyper-Heuristics

During the optimization processes they perform, generation hyper-heuristics dynamically generate new heuristics or modify pre-existing ones. Contrary to selection hyper-heuristics that perform their search in a search space containing complete pre-existing heuristics, generation hyper-heuristics perform their search in a search space consisting of heuristic components. In this manner, generation hyper-heuristics create new heuristics through the use of the algorithm components. This goal can be achieved in various manners, such as using machine learning methodologies, genetic programming (an evolutionary approach that “genetically breeds” a population of computer programs in an artificial, evolution-like manner by transforming the existing ones in an iterative manner) [173] and search-based techniques.

### 5.3. Ensemble Hyper-Heuristics

The idea of ensemble hyper-heuristics is to make a combination of two or more lower-level procedures or algorithms to generate a novel algorithm that will cover a number of different strategies for finding the solution to the problem in a variety of situations. Combining diverse heuristic/metaheuristic algorithms, i.e., using an ensemble of optimization strategies, means uniting their strengths and combining their results to arrive at a novel and improved method of solution. This approach is related to generation hyper-heuristics.

The techniques used to combine outputs of such ensemble sets of algorithms include the method of weighted averages, voting, artificial neural networks and some other machine learning methods. In this way, a generalized intelligently combined procedure is obtained that functions better than a sum of its parts (i.e., better than any of these sub-procedures or sub-algorithms operating independently).

## 6. Hybridization Methods

One of the relatively often used approaches in bio-inspired optimization is the hybridization of two or more different techniques, each with its own advantages and disadvantages, in order to boost their advantages and to lessen or even cancel disadvantages. In order to belong to the main topic of this survey, at least one of them should be biomimetic. Hybrid approaches make use of the complementary strengths of the combined methods. In this way, the solution quality and accuracy are enhanced, and the efficiency and robustness of the resulting strategies are improved over their constitutive blocks. In this way, an effective and flexible and effective method to solve complex optimization problems is obtained. The choice of hybridization type will be dependent on the particular problem, as well as the required optimization objectives.

**Figure 25:**
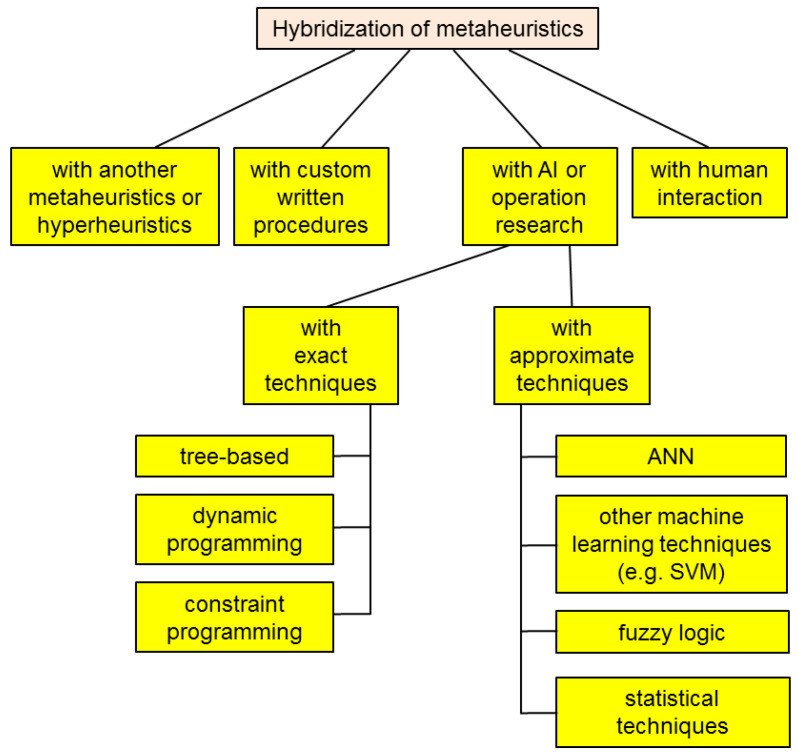
Metaheuristic hybridization method classification according to [174], but somewhat modified and extended.

The subject of hybrid metaheuristics is almost a separate science field in itself. For an excellent overview of its methods, taxonomy and approaches, see [174]. Figure 25 shows a possible classification of the hybridization methods, based on the mentioned reference by Raidl, but somewhat modified. The classification is by no means exhaustive, and it could be extended to include more methods.

Numerous treatises dedicated to hybridization methods have been written. For the sake of completeness, we mention some selected details on them. However, we must restrict ourselves here, being aware that we are only scratching the surface. The further text gives a very short overview of the most pertinent bio-inspired hybridization methods.

### 6.1. Hybrids of Two or More Metaheuristic Algorithms

The most obvious method to perform hybridization is surely to combine two or more metaheuristic algorithms into a single hybrid [175]. One of the possible approaches to this task is switching between separate algorithmic methods at various points of the process of bio-inspired optimization. For instance, a hybrid could commence using a global algorithm for general exploration of the search space, and then switch to a local search algorithm to explore the immediate vicinity of a solution discovered by the global algorithm and thus refine that solution. In this way, merging of two metaheuristic results in a hybrid that retains the advantages of both separate constituents. The applicable global search algorithms for this purpose include particle swarm optimization and other globally oriented swarm intelligence algorithms, or one may apply evolutionary algorithms such as a genetic algorithm, memetic algorithm or differential evolution algorithm. Local search can be then accomplished using some non-bio-inspired algorithms, e.g., hill climbing or the very popular simulated annealing. In this way, both exploration and exploitation are boosted compared to a single-algorithm case.

An alternative approach to the hybridization of metaheuristic algorithms would be to use ensemble methods that utilize two or more metaheuristic algorithms that would function independently of each other. These independently determined solutions can be combined by averaging or weighted aggregation. Such an approach offers the benefit of diversity of the optimization search processes and thus contributes to increased accuracy.

Hybrids between two metaheuristic algorithms have been successfully applied in microelectronics optimization [176] (whale optimization algorithm-particle swarm optimization) and in nanophotonics optimization [177] (gravitational search algorithm-particle swarm optimization).

### 6.2. Hybrids with Hyper-Heuristics

Hybridization of metaheuristics with hyper-heuristics enables automation or even the generation of custom heuristics tailored for the investigated optimization case [178]. As said before, hyper-heuristics are able to adaptively choose or even create convenient heuristics customized to a given optimization problem according to its properties and performance. This ensures additional flexibility when optimizing a particular problem.

### 6.3. Hybrids with Mathematical Programming (MP)

Mathematical programming techniques can be incorporated into a biomimetic metaheuristic algorithm to ensure a fully accurate and precise solution of specific problems that appear as subunits of the metaheuristic algorithm instead of using heuristic ones [179]. This will boost the overall accuracy of the solutions since some parts/stages of the algorithm will be solved using an exact approach instead of heuristics. In this manner, the strengths of both approaches will be combined to offer a better solution. 

An exact approach that can be used includes the utilization of linear programming or integer programming to calculate a better initial solution. Thus, exact calculation is used to improve initialization and in that manner to increase the chances of arriving at a better final solution in a shorter time.

Other cases of the use of mathematical programming are hybrids of metaheuristic algorithms with constraint programming which enable dealing with complex constraints, because constraint programming enables one to perform modeling problems with constraints and to solve them [179]. In constraint programming, one declaratively states the constraints to a set of decisive variables which determine the feasible solutions. Constraints are defined as relations among multiple variables. These relations pose the limits to the values the said variables can have at the same time. While the metaheuristic algorithm optimizes the objective function, the constraint programming deals with the constraints, thus ensuring the feasibility of solutions, making them more robust.

Local search integration can be incorporated into metaheuristic exploration of the search space during the stage of local search. In this way, mathematical programming is used to calculate the neighbor solution. MP can be custom-written for specific problem structures to exploit their properties that can lead to simpler or faster and more accurate solutions.

Mathematical programming in general can be used to refine solutions already obtained by metaheuristics and further optimize them. It could be accomplished for instance by relaxing some constraints, introducing additional objectives or adjusting the variables for decision making.

### 6.4. Hybrids with Machine Learning Techniques

There is a plethora of machine learning techniques, and many of them can be incorporated into metaheuristic frameworks to improve or guide the exploration of the solution space. Machine learning itself [180] is a huge field of its own. Obviously, its many techniques can boost and enrich metaheuristics. Machine learning can be used for training that will be able to predict promising areas of the search space so that metaheuristics can avoid a large part of the exploratory operation and concentrate instead on the zones where the best solutions are already expected. Thus, the convergence speed can be improved. The rest of this subsection handles some of the machine learning techniques most often hybridized with metaheuristic algorithms. The interesting point relating this topic to the main goal of this review is that these techniques themselves are loosely bio-inspired.

Artificial neural networks are one of the most important machine learning techniques that are used to enhance metaheuristic algorithms. A separate subsection in this review is dedicated to this type of improvement, and because of that, no further description will be given here.

Support vector machines (SVMs) also belong to machine learning techniques that are sometimes used hybridized with metaheuristics. SVMs represent a supervised learning technique that incorporates learning procedures convenient for regression analysis and data classification. Metaheuristics can be used to determine the optimum values of parameters of support vector machines, such as their regularization factors or kernel parameters. In this way, the optimum configuration within the SVM hyperparameter space is found, and the support vector machine offers its maximum performance for the investigated dataset.

### 6.5. Hybrids with Fuzzy Logic

If the optimization problem contains uncertainties of vague elements, it is convenient to incorporate fuzzy logic into the metaheuristic algorithm [181]. Fuzzy logic will be able to guide the metaheuristic search process by providing membership functions and linguistic rules which will serve to direct the search process in vague situations. Thus, both exploration and exploitation can be improved.

## 7. Multi-Objective Optimization (MOO)

The vast majority of the algorithms presented so far in this text are single-objective algorithms, meaning that they have a single objective function, their solution space is unimodal (with a single optimal solution or a global optimum) and their algorithms tend to be simpler and computationally less demanding. However, many practical problems come with more than one objective function that should be optimized at the same time, typically with conflicting requests. The approach dedicated to their solving is multi-objective optimization or multi-criteria optimization. Other names found in the literature for this method are vector optimization, Pareto optimization and multi-attribute optimization.

Contrary to single-objective algorithms, MOO algorithms have two or more conflicting/competing objectives, and there are multiple objective functions that have to be simultaneously optimized. Their solution space is either non-unimodal or multimodal. Since multi-objective optimization considers multiple objectives simultaneously, instead of finding an optimal or near-optimal solution as in optimization related to a single-objective function, it rather aims to find a set of solutions that achieve a trade-off between these objectives. The set of solutions that represent the best possible trade-offs among the conflicting objectives is called the Pareto front or Pareto set. A solution is considered Pareto-optimal if there is no other solution that can improve one objective without worsening at least one other objective, if there is no other feasible solution that dominates it (a solution A dominates another solution B if it performs better in at least one objective without being worse in any other objective). This means that the Pareto set is the set of all Pareto-optimal solutions in the objective space, i.e., the set of all non-dominated solutions. The algorithm aims to find a set of solutions that covers this front. Multi-objective algorithms are generally more complex due to the need to handle multiple objectives and to explore the whole Pareto front. In the case of MOO, the decision making is more complicated since there is no single best solution. The decision maker must take into account all of the trade-offs between conflicting objectives and make informed decisions based on personal preferences or domain-specific criteria.

There are serious challenges with MOO. Among them is the problem of high dimensionality. The mathematical and computational complexity of the problem exponentially increases with the number of variables, and this becomes very serious for large-scale problems. Thus, a thorough exploration of the solution space becomes exceedingly difficult. Finding and representing the Pareto front as accurately as possible is a significant challenge in multi-objective optimization since it requires extensive exploration of the solution space. This results in another closely connected challenge, that of large computational complexity: the solutions of multi-objective algorithms are generally vastly more computationally demanding than those for single-objective ones. The main challenge of MOO lies in finding a balance among numerous conflicting objectives and determining the right trade-offs. The challenge is further aggravated because these both problems are subjective and depend on the preferences of the decision maker, a situation that may be problematic, to say the least, and is definitely difficult to quantify in any meaningful way. Yet another problem is the need for tools and methods to help the decision maker in the decision, especially those for the visual presentation of data in multidimensional spaces. Further, generation of a set of different solutions that adequately and without redundancy covers the entire Pareto front can be challenging. This generation usually requires special tweaking of algorithms and even the generation of new ones, either customized to the problem at hand or created as hybrids of two or more software approaches (see Section 6).

The question of choice between single- or multiple-objective (or hybrid) algorithms reduces to the question of the number of conflicting objectives posed by the problem. However, if there is an obvious, recognizable and relatively easily resolved trade-off among multiple objectives, then the decision maker might decide to utilize a single-objective approach in spite of the problem having more than one objective. This reduces to the question of the complexity of the problem: if it is not possible to make a decision regarding the trade-off, or if the structure of the problem is non-unimodal or multimodal, then the MOO is more appropriate. Another decision factor is determined by the available computational resources and the time constraints, bearing in mind that MOO is highly demanding for both. Finally, the subjective personal preferences of the decision maker will often tip the balance toward one of the available options.

Bio-inspired multi-objective optimization methods can be based on the application of procedures such as metaheuristic algorithms or neural networks and generally AI. The next three subsections consider the main groups of MOO methods.

### 7.1. Multi-Objective Metaheuristics

Commonly used in multi-objective optimization are metaheuristic evolutionary algorithms such as genetic algorithms, memetic algorithms or differential evolution, but particle swarm optimization, artificial immune systems, ant colony optimization and other metaheuristic algorithms are also used due to their ability to explore the solution space and find diverse Pareto-optimal solutions, which represent the trade-off between different objectives. Many single-objective metaheuristic algorithms can be adapted and extended to solve multi-objective problems. 

The area of metaheuristics-based multi-objective optimization is enormously vast. In [182], it is mentioned that the EMOO repository (its web address is given in the reference quoted in the previous sentence), related solely to a single type of multi-objective optimization (the evolutionary algorithms), contains over 12,400 bibliographic references alone (publications and software).

A book on evolutionary algorithms for solving multi-objective problems by Coello Coello, Lamont and Veldhuizen [183] offers a much deeper insight into the subject. It gives a taxonomy of multi-objective optimization methods together with detailed descriptions of each of them and a comprehensive coverage of applications.

Table 7 lists some metaheuristics-based multi-objective optimization algorithms used for applications in the fields of microelectronics and photonics including sensorics, telecommunication and fog/cloud computing. The choice of algorithm depends on the problem characteristics, available resources, and specific optimization requirements. Each algorithm brings its own set of advantages, making it suitable for different problem domains and optimization requirements.

Besides using metaheuristic algorithms, multi-objective optimization can be implemented using machine learning techniques such as artificial neural networks (multi-layer perceptrons), convolutional neural networks and recurrent neural networks.

### 7.2. Machine Learning in Multi-Objective Optimization

The term “learning” does have its roots in biological behavior, and machine learning (ML) does belong to the wider field of artificial intelligence. However, ML is a very broad term, and besides some biomimetic procedures that it includes (e.g., neural networks), it also encompasses a number of other methods that are not bio-inspired (a typical example would be support vector machines). Most of the non-biomimetic methods are based on mathematical approaches such as information theory, statistics or different exact optimization techniques. We include general ML here briefly because it partially consists of procedures that do belong to biomimetics, because it is a subfield of the much wider area of AI and, finally, for the sake of completeness, comprehensiveness and self-containment of this text.

The following subdivisions of this subsection very briefly outline some ML methods most often met in multi-objective optimization. The list is illustrative, not exhaustive, and the methods are presented in no particular order.

#### 7.2.1. Neural Networks in Multi-Objective Optimization

Neural networks belong to biomimetic algorithms, especially artificial neural networks. They have been used for multi-objective optimization [205]. Because of their importance in both machine learning and multi-objective optimization, we describe them in more detail in Section 8. The artificial neural networks themselves are dealt with in Section 8.1, which is dedicated solely to them.

#### 7.2.2. Surrogate Models in Multi-Objective Optimization

Also known as response surface models, emulators, metamodels or approximation models, the surrogate models are designed to approximately mimic in a computationally less expensive manner those parts of the optimization problem that are not easily determined. They are used in multi-objective optimization to approximate (make surrogates of) the objective functions or constraints within an optimization problem. By training them on a set of pre-determined solutions, one can utilize them to predict approximate values of objectives in the parts of the search space that still remain unexplored. This can greatly help to reduce the computational burden in complex multi-objective environments. Their use in multi-objective optimization was considered in [206] for the case where there are uncertainty problems.

#### 7.2.3. Reinforcement Learning in Multi-Objective Optimization

Reinforcement learning algorithms represent a computational approach to learning that encompasses methods of performance improvement using a trial-and-error approach with resulting rewards or penalties; i.e., they represent learning without a training dataset and only utilize training based on action. Based on experience from these actions, they determine which action results in the greatest reward. Some authors [207] write that their roots are in experimental psychology, which would make this algorithm basically bio-inspired. Reinforcement learning algorithms learn to select those decision variables in multi-objective problems that can perform optimization of multiple objectives at the same time. Methods of reinforcement learning adopted for multi-objective optimization include policy gradients, Q-learning, Monte Carlo and the temporal difference learning methods (not to be further handled within this text). In such an action-based learning way, the search for a Pareto-optimal solution is performed. Some publications related to reinforcement learning in multi-objective optimization include [208,209]. Metaheuristic algorithms such as evolutionary algorithms may be hybridized with reinforcement learning in order to guide the exploration of the search space towards the Pareto-optimal solutions.

#### 7.2.4. Gaussian Processes in Multi-Objective Optimization

A Gaussian process (GP) is a stochastic process designated by its mean and covariance functions. Gaussian processes generally represent probabilistic methods applicable, e.g., for regression problems.

Gaussian processes can be of great use in multi-objective optimization [210]. However, it is important that they do not represent optimization algorithms by themselves. They are actually modeling tools typically used to generate surrogate models. These surrogates can then be incorporated into metaheuristic algorithms such as evolutionary algorithms or particle swarm optimization algorithms. The usefulness of GPs in surrogate models is reflected in their capability to capture nonlinear relationships. This makes them convenient for modeling complex objective functions. 

They constitute a probabilistic framework that gives estimates of the values of the objectives and includes their uncertainties. By their nature, Gaussian processes are most useful when handling noisy or limited datasets. This property makes them convenient for quantifying uncertainty in multi-objective optimization procedures. In that manner, one can assess the confidence in the prediction of the Pareto front or identify high-uncertainty regions of the input space. This is very convenient for guiding exploration during the search process and estimating the Pareto front.

Decision makers can readily incorporate their preferences (including constraints), thus making GPs inclined towards the regions of search space that are desirable for decision makers and targeted by them because they satisfy some specific requirements.

A GP can be are also used in reinforcement learning in several different manners. It can be trained on observed state-action pairs and their corresponding next states (rewards), and based on this it can be used to predict the following reward. Besides that, a GP can also give estimated uncertainties for its predictions of reward, which is useful during exploration as it can lead to exploring some uncertain regions and thus learning more about the search space. The knowledge of uncertainties can be used to assess the robustness of the overarching algorithm and its safety in situations where erroneous choices could cause hazards or damages. Finally, a GP can be used in the traditional manner to calculate approximations of functions used in reinforcement learning.

The application of Gaussian processes in multi-objective optimization is investigated in [211]. The authors used GPs to generate a surrogate model and used it in further training.

## 8. Neural Networks and Multi-Objective Optimization

Most neural networks (NNs) are general computational models belonging to AI; as such, they themselves do not belong to bio-inspired optimization algorithms, except in name. However, even such types of NNs can be used together with bio-inspired optimization algorithms as a combination or hybrid. Some types of neural networks do belong to biomimetic procedures. Many of the NNs can be utilized in multi-objective optimization, in situations where traditional approaches meet serious problems due to the high dimensionality and the need to reach trade-offs, which is naturally followed by the vastly increased computational complexity of the problems. The following text handles some cases of neural networks being useful for multi-objective optimization.

### 8.1. Artificial Neural Networks

**Figure 26:**
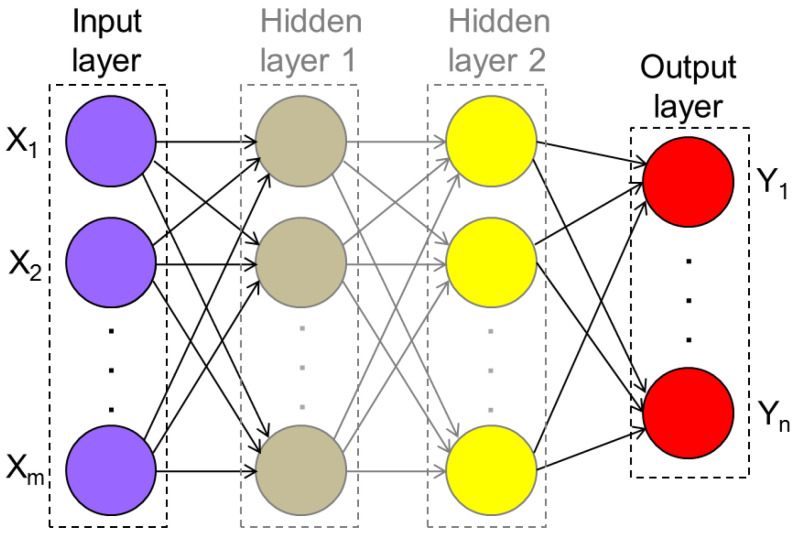
General structural diagram of a feed-forward ANN with *m* inputs, *n* outputs and more than one hidden layer.

Artificial neural networks (ANNs) are computational models, biomimetic by their structure and their modes of operation. They represent the foundational neural network algorithms. They are inspired by the structure and function of the central nervous systems of living organisms, in particular of biological networks of neurons in the brain. An example is an ANN where a network of “synaptic-like” connections, “neurons” (nodes of the network), is organized in layers, as shown in Figure 26.

Depending on the number of layers, ANNs can be shallow (one layer hidden between the input and output layer) or deep (more than one hidden layer). In the brains of living organisms, neurons may not be organized in layers, and in the presented ANN structure there are no “synaptic” connections between neurons in a single layer. However, analogously to neural firing, signals from neurons positioned in one layer spread simultaneously to neurons positioned in the subsequent layer. Such a system of interconnected nodes (“neurons”) is capable of machine learning and pattern recognition.

Besides the structure of ANNs being bio-inspired, the very manner in which ANNs function/operate is also biomimetic. In every node, a function of a perceptron is performed (weighted and biased sum of inputs, activated by some activation function-like step, linear or sigmoidal). In the process of creating an ANN, the weights and biases of its nodes are iteratively altered based on the network’s prediction errors; the network evolves, and its evolution is called learning. It may be supervised, unsupervised or reinforcement learning. The learning is supervised if the network is fed by examples aimed as a guide for tracking the difference between the instantaneous values predicted by the network and the correct values valid for the same set of input parameters. It is unsupervised otherwise. Reinforcement learning, also called approximate dynamic programming or neuro-dynamic programming [212], is learning where the focus is moved from examining the individual examples to the effect of the decision outcome on the whole environment and the possibility to gain the cumulative reward. Many network parameters, such as its architecture, biases, weights and connections of neural network nodes, directly affect the network’s performance metrics such as accuracy and convergence speed.

Apart from being biomimetic per se, artificial neural networks incorporate optimization algorithms which are often biomimetic; i.e., they are used in hybridized solutions. Some examples of the use of biomimetic optimization algorithms such as genetic algorithms (GAs), ant colony optimization (ACO), differential evolution (DE) or particle swarm optimization (PSO) for the optimization of performances of artificial neural networks are reported in [213,214,215].

The principles of creating ANNs are based on using iterative processes to converge to solutions that minimize an objective function. That means that ANNs can serve as optimization algorithms themselves. As reported in [216], one variant of artificial neural networks, the Hopfield network, can be used to solve a constrained least squares optimization problem, which has been demonstrated in adjusting filter parameters in digital signal processing.

Boltzmann machines, also a variant of artificial neural networks, have been integrated in the estimation of distribution algorithms and used in solving combinatorial optimization problems with a single objective [217]. In [218], a population of Boltzmann machines, along with the particle swarm optimization rule aimed to re-initialize the neuronal states of Boltzmann machines upon their local convergence to escape from local minima toward global solutions, was used for Boolean matrix factorization.

Another way of ANNs being a part of an optimization algorithm is when ANNs are used as fitting functions. Many phenomena, including some related to the design of microelectronic and nanophotonic devices, cannot be modeled by explicit mathematical functions. Complex models can be optimized more easily if we employ ANNs obtained by training on a subset of the objective function evaluations. ANNs can predict the objective function value for unexplored regions of the search space. As surrogate models, computationally efficient approximations of complex functions, ANNs enable faster and more efficient exploration of the optimization landscape during the optimization process.

Similarly to training ANNs on examples related to objective functions that need to be optimized, ANNs can be trained on various combinations of model performance and parameter settings of other machine learning algorithms and used to optimize their convergence and for hyperparameter tuning [219,220].

Table 8 summarizes the roles of ANNs within the context of bio-inspired optimization. This also includes the optimization of ANN parameters by metaheuristic methods. Only some cases of the latter are presented as an illustration, and the list is far from being exhaustive. A review of the use of metaheuristics for ANN optimization can be found in [221].

### 8.2. Convolutional Neural Networks (CNNs)

Convolutional neural networks represent one of the few basic architectures used in deep learning. The primary fields of application of CNNs are image analysis and recognition, segmentation and object detection. Their use in multi-objective optimization is also known. Since CNNs are convenient for dealing with images and patterns, they can be used in those multi-objective problems where objectives or decision variables resemble images, i.e., whose structure is spatially variable. CNNs are convenient for recognizing patterns in the input information.

Their applicability is high in image-based multi-objective optimization. Some optimization problems include solutions or decision variables that are representable as images or possibly grids of pixels varying in dependence on the region of the object. A CNN can analyze these images in its usual manner, thus ensuring the recognition of differences between various areas of the search space. Such problems are encountered, e.g., in layouts of microelectronic and all-optical nanophotonic circuits, as well as in other related fields. For example, in spatial optimization problems, the solutions can be represented as pixel values corresponding to different regions or objects. CNNs can be applied to process and analyze these images, enabling the optimization process to leverage the spatial information and relationships between different regions or objects. This approach has been used in applications such as land-use optimization, urban planning and material design.

Other applications of CNNs in multi-objective optimization include their use as surrogate models in order to approximate the objective functions, in extracting compact representations of the decision variables that not only contain relevant information on variables but also contain important correlations between them. This may reduce the dimensionality of the search problem and thus speed up exploration. Another benefit is the possibility to extract relevant features from complex datasets. Thus, extracted properties can be then fed into standard multi-objective procedures and algorithms and thus further improve the search. An example of the use of CNNs for multi-objective optimization is given in [222].

### 8.3. Recurrent Neural Networks (RNNs)

RNNs are neural networks convenient for handling time-dependent or sequential data. When utilized for multi-objective optimization, they can be applied to model dynamic optimization problems or time-dependent problems. They are able to learn the history of the optimization process and its context. Similar to CNNs, RNNs can be utilized as surrogate models to be built into traditional multi-objective algorithms to facilitate the exploration of the Pareto front. They utilize the recurrent connections in the learned map between the input parameters and the objective values that correspond to them. In this way, they form relationships between different time steps. They can also learn to generate the next set of decisions based on the past decisions and their results. In this way, an RNN makes use of the memory of the past decisions and their outcomes and helps improve the exploration of the search space. RNNs tend to be oriented towards a particular problem, and a versatile network of this kind that would be able to solve a wide range of optimization problems represents a very difficult problem. The paper [223] described a recurrent neural network aimed at the solution of a class of generalized convex optimization problems.

### 8.4. Radial Basis Function (RBF) Networks

RBF networks are built with an input layer, an output layer and one or more hidden layers having radial basis function units. In multi-objective optimization, these neural networks, similarly to a number of other NNs, are used to generate surrogate models that can serve as approximations for objective functions. They are able to generate a nonlinear mapping between the decision variables and the objectives. They can be combined with metaheuristic algorithms such as swarm intelligence or evolutionary algorithms and thus be used for the ensemble approach. RBF networks can be applied to generate new candidate solutions, propose new configurations of parameters and approximate complex relationships. In that manner, they help explore the decision space in multi-objective optimization [224,225].

### 8.5. Generative Adversarial Networks (GANs)

These neural networks actually consist of two networks, a generator and a discriminator, that compete against each other. The generator network generates candidate solutions, and the discriminator network evaluates the fitness of the solutions. When used for multi-objective optimization, GANs generate Pareto-optimal solutions with high quality and enhance their diversity.

GANs are also used to generate surrogate models: the generator maps the latent space vectors to corresponding objective values, thus ensuring predictions for not-yet-seen parameters and parameter configurations. Some subgroups of GAN are the original GAN (unchanged, in the form as proposed for the first time in 2014, also sometimes called the Vanilla GAN), Wasserstein GAN (a modified version of Vanilla, offering increased training stability and avoiding mode collapse), conditional GAN (a modified Vanilla that includes additional information, i.e., auxiliary attributes and constraints), deep convolutional GAN (it introduces convolutional neural networks (CNNs) into both the generator and discriminator, useful for image processing and optimization).

The use of generative adversarial networks for the optimization of active metasurfaces based on transparent conducting oxides was presented in [226]. It applied an approach that represented a combination of the K-means clustering algorithm and a conditional Wasserstein GAN.

### 8.6. Autoencoders

Standard autoencoder networks are unsupervised neural networks that are able to learn compact representations of the input data by encoding them into a lower-dimensional latent space. They consist of an encoder network that performed the mentioned compacting and a decoder network that performs the opposite and reconstructs the input data from the latent space. When used in multi-objective optimization, autoencoders utilize the reduced dimensionality of the design space for the simplification of the optimization process. Their other role is that they use the mentioned learned representations to enhance the optimization procedure. They are also used to generate surrogate models based on the relationships between the input data and the objective space. Besides the standard form, there are several versions of autoencoder networks such as the adversarial autoencoder (includes some properties of generative adversarial networks and, besides the encoder and decoder, also contains a discriminator network), variational autoencoder (includes an additional regularization objective, encodes the input data into a mean and variance parametrization), denoising autoencoder (reconstructs data to clean them from noise and perturbations) and sparse autoencoder (introduces sparsity constraints and thus activates a small number of neurons in the latent representation). An application of a variational autoencoder network in optimization is presented in [227] where the autoencoder is hybridized with a particle swarm metaheuristic algorithm.

## 9. Applications in Microelectronics

One of the applications of bio-inspired methods is their use in the design of microelectronic and nanoelectronic circuits. Various circuit parameters can be optimized, including the properties of the single circuit elements, circuit floor plan, layout, routing of the interconnects, sizing and many more. Since it is often necessary to simultaneously optimize more than a single parameter, multi-objective bio-inspired methods are used in such situations. Typical optimization tasks achievable by bio-inspired algorithms include the following:

- Circuit Element Parameters [228]: The properties of the components built into a microelectronic circuit can be optimized in order to achieve targeted circuit performance, e.g., desired speed, power consumption, decreased heat dissipation and decreased noise. The values of passive device parameters such as resistances, capacitances and inductances [228] and also various parameters of active devices (different types of transistors, amplifiers, analog-digital converters, etc.) are optimized.
- Floor Planning and Topology [229,230]: This crucial optimization step includes the placement of the circuit elements on a chip and the routing of their interconnects. This helps minimize crosstalk, avoid breakdowns, minimize leakage currents, ensure more homogeneous heat dissipation, etc.
- Circuit Sizing [231]: Circuit area minimization is critical for practically all microelectronic devices and systems, especially for implantable and wearable healthcare devices and generally those where the area of the circuit is limited by design requirements. Optimal circuit sizing is actually of interest for basically all microelectronic circuits since it helps improve overall performance and enhance circuit reliability.
- Power Consumption [229]: Bio-inspired algorithms can be employed to optimize power consumption by minimizing leakage current, optimizing voltage levels and reducing dynamic power dissipation in circuits. This directly improves circuit reliability by avoiding overheating, and it also helps in keeping the power consumption at its minimum, which is of paramount importance for all battery-supplied circuitry.
- Sensitivity to Design Parameter Variations/Robustness [232]: Optimizing designs to wider variations in process parameters (temperature, atmosphere, material choice and the tolerances of their properties, various technological uncertainties) can be used to achieve the maximum performance robustness and insensitivity to variations in external parameters.
- Production Yield [233]: Bio-inspired optimization can help decrease faults and increase the percentage of successfully produced chips during planar technology fabrication. By considering process variations, temperature variations and component tolerances, bio-inspired algorithms can optimize circuit designs to achieve robust performance and improve yield.

All of the above can be used for all types of microelectronic circuits, e.g., analog [234], digital [235] and mixed-signal type [236]. Many of the quoted optimization tasks can be also applied to microelectromechanical systems (MEMSs) as well [29,237]. The further two subsections present two important optimization procedures as case studies in more detail.

### 9.1. Optimizing Analog Circuit Sizing

**Figure 27:**
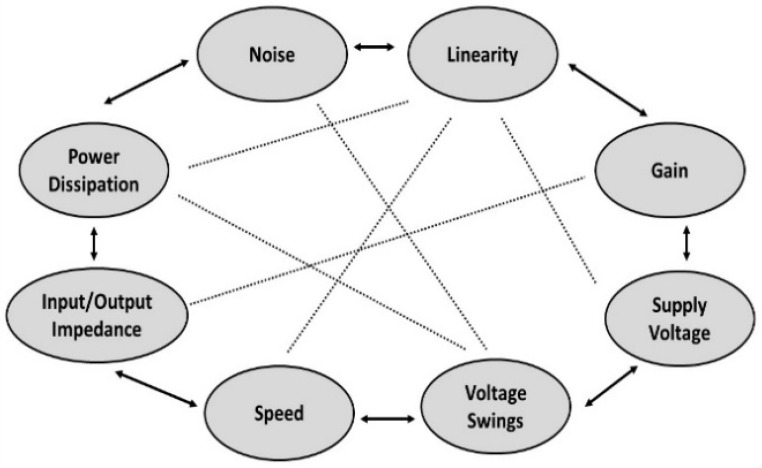
Analog design octagon: trade-offs in analog circuit design.

The design of analog integrated circuits, although it had its heyday some decades ago, retained its secure place in various fields of microelectronics [238]. We consider here the operational transconductances (OTAs) where the aspect ratio of their MOS transistors should be appropriately sized to accomplish the required design. The sizing of transistors manually is a tedious process that increases the complexity and is time-consuming. This constraint demands a reliable method to attain the transistor sizing by balancing all the desired parameters of the design. The use of metaheuristic optimization algorithms to determine the transistor sizes can be a solution to this problem (e.g., [239,240]). The minimization of the parameters can be accomplished by selecting one or more objective functions and considering the rest of the parameters as a constraint. Wide research has been performed on this topic, and researchers have proposed methods of optimization to use in circuit designs for better sizing and biasing of the devices. PSO and its variations have been largely utilized in automated circuit sizing over the years [236]. In analog design, there is a trade-off among parameters, as seen from the analog design octagon [238] shown in Figure 27.

There are many quotations of bio-inspired techniques in the literature within the context of microelectronic circuit optimization. For instance, Jassimet et al. [241] and Fakhfakh et al. [242] used PSO to minimize power and for the optimization of both mono-objective and multi-objective functions in a high-frequency low-noise amplifier with a biasing network. Barari et al. [243] and Rojec et al. [244] used evolutionary algorithms to design analog circuits. Barari et al. used a combination of the metaheuristic PSO and the evolutionary genetic algorithm (GA) to design a two-stage op-amp, with figure of merit as the objective function. Rojec et al. proposed a novel approach to analog circuit optimization capable of performing optimization not only in the parameter space, but also in the topological space.

Even though the PSO and its variants have advantages over other algorithms in analog circuit design, they are computationally inefficient and have a slow convergence speed. Most of the variants have failed to find a global optimum solution to complex mathematical and real-life problems, especially in the case of multi-objective functions, where there are multiple local minima. Dendouga et al. [245] proposed a multi-objective genetic algorithm for the minimization of the area and maximization of the unity gain bandwidth in a two-stage op-amp.

There are other works implementing different algorithms with the same goal as those quoted above. Kudikala et al. [246] used the harmony search algorithm (HS) and differential evolution (DE) algorithm for the minimization of errors in a folded cascode OTA. The convergence speed of the HS is comparatively higher, but it fails to converge to global optima. Majeed et al. [247] used the grey wolf optimization (GWO) technique to minimize the area offsets in two analog circuits, a CMOS differential amplifier and a two-stage CMOS op-amp. They have proven that for these particular applications, their approach shows superiority over some other competing algorithms such as the PSO and a non-bio-inspired technique, the gravitational search algorithm (GSAPSO), the main advantage being a higher GWO convergence speed over that of PSO and GSAPSO. Majeed et al. [248] also used a hybrid approach, that of the whale optimization method and the grey wolf optimization, to minimize the area of a two-stage op-amp.

**Figure 28:**
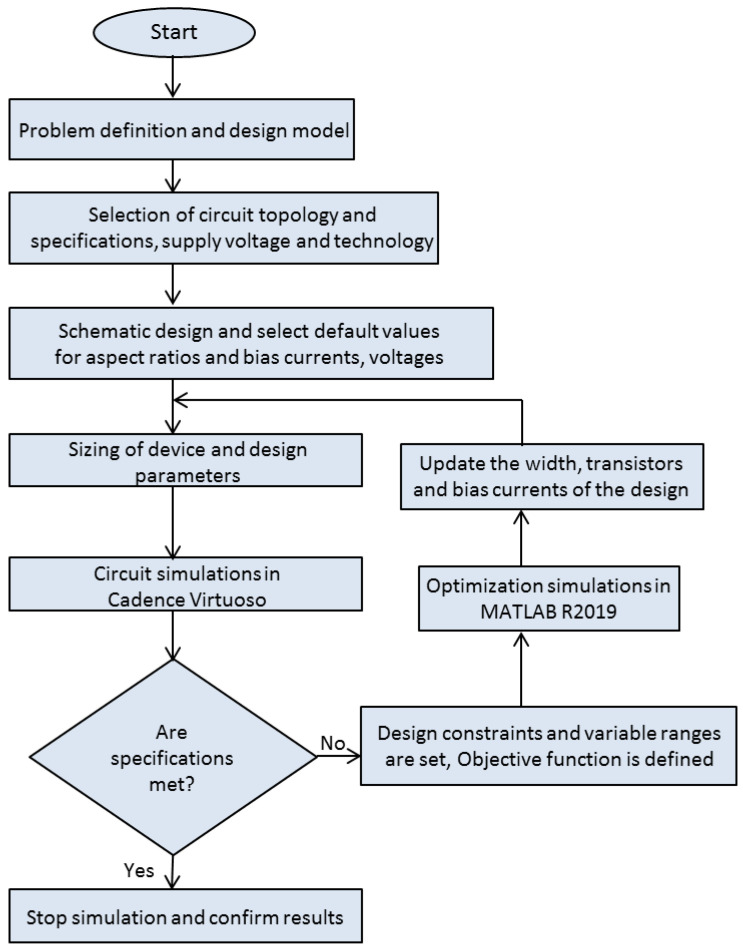
Flowchart of a particular application of metaheuristic approach in analog circuit optimization [231].

The trade-offs in analog circuits which are considered multi-objective problems can be solved with bio-inspired methods. Some examples include the publication of Bachir et al. [249] where attempts were made to solve a multi-objective problem in a two-stage operational amplifier using multi-objective ACO. The two objective functions taken into consideration were the minimization of the die area and the power consumed. In a similar work by Benhala [250] dedicated to the design of a two-stage op-amp with a minimized area and a maximized common mode rejection ratio, the authors concluded that the use of the ant colony optimization method shows a significantly lower convergence speed and increased computing time in comparison to their proposed combination of the backtracking search and ACO (BAACO) for the design of a two-stage op-amp with a minimized area and a maximized CMRR. An exemplary flowchart for a particular complete circuit design of a self-biased amplifier using metaheuristic methods is shown in Figure 28 [231].

### 9.2. Optimizing Circuit Routing

Researchers use techniques including the sequential A* method, Lee’s algorithm and standard MCTS approaches to resolve circuit routing problems [251]. In building electronic systems such as integrated circuits (ICs) and printed semiconductor boards (PCBs), circuit routing is the key design challenge. Circuit routing creates patterns of lines for connecting contacts or terminals of circuit components, akin to a way of establishing paths connecting two places. It is a difficult task because a very vast search space is involved in locating routes between the densely packed and relatively large electronic devices. Current remedies have been deliberately created using domain expertise to certain design rules, making it challenging to adjust them to newly emerging issues or design requirements. A universal routing strategy is therefore greatly desirable [252]. Two-stage approaches, global routing followed by detailed routing, force-directed routing, region-wise routing, and rip-up and reroute [253,254,255], have been proposed to resolve complex large-scale circuit designs and to analyze track congestion at the global routing stage.

A deep Q-network (DQN) is used in a new study that analyzes worldwide routing as a deep reinforcement learning (DRL) problem. The DQN makes a choice based solely on the output of its policy, as opposed to the deep neural network (DNN)-based MCTS (Monte Carlo tree search), which bases decisions on the outcomes of numerous simulations utilizing an established policy. An unknown complicated circuit cannot necessarily be solved satisfactorily by a trained DQN policy [256]. Given the outcomes of global routing, detailed routing creates interconnects with the appropriate shapes and placements. The channel routing algorithm is among the most widely used ones. The routing region is typically divided into routing channels, and the wires are connected within these channels [257].

According to some researchers, global routing and detailed routing do not work together well for complicated routing problems, and when this two-stage strategy is used, it typically leads to complex software systems that are challenging to upkeep and to a meandering workflow that hinders the creation of circuits. In contrast to the two-stage method, in a method known as area routing, routes are typically built directly for the designs with just a handful of layers of metal and a limited number of nets [258].

### 9.3. Future Directions

Challenges that define future directions of bio-inspired optimization algorithms in research and practical implementation in microelectronics include the implementation of multi-objective optimization and hybridization. These two fields are of general interest since they are able to define the general directions of future development of biomimetic optimization algorithms. One of the important directions of future research is determined by the challenge of how to ensure scalability of the optimization algorithms with ever-increasing complexity and sophistication of microelectronic and nanoelectronic circuitry and inclusion of multitudinous constraints. This is directly related to the need for computationally cost-effective optimization solutions, which are also one of the important general challenges nowadays.

One of the likely directions of future research is the design of integrated circuitry for hardware implementation of optimization algorithms. This should ensure facilitated and accelerated implementation of complex optimization procedures. An important avenue of future research in AI-optimized microelectronic circuitry is how to provide increasingly robust solutions, able to tackle the dynamic and constantly evolving environment of microelectronics development. It is a known fact that hardware specifications permanently evolve to keep up with Moore’s law. Similar things are happening with the system complexity and sophistication of production technologies. Thus, the optimization algorithms must be prepared for this. So far, the prospects appear bright in all of the quoted areas.

## 10. Applications in Nanophotonics

Nanophotonics represents the investigation of materials and devices with the dimensions of their characteristic features or building blocks in the range of the order of hundreds of nanometers or below, down to the dimensions of a few nanometers. A vast number of such structures have been proposed, often exhibiting properties that appear counterintuitive, such as negative or near-zero effective refractive index, cloaking properties (“invisibility cloaks”), perfect mirroring in 3D, superabsorption and resolution far below the diffraction limit, to name just a few.

Optimizing nanophotonics has been traditionally accomplished using intuition-based strategies. That is to say, new devices were developed in such a manner that first a new physical effect would be observed in a nanophotonic structure, and then its properties would be matched to convenient applications. Optimizations would be accomplished by tuning a chosen subset of structural parameters, typically for the simplest geometries and layouts. For that, the trial-and-error strategy and the parameter sweep method were typically utilized.

The ever-increasing complexity of the targeted structures and devices demanded a new approach that would be able to simultaneously optimize a larger number of parameters. An approach to resolve this is the inverse design, where the designer starts from a desired set of targeted properties of the final product and then returns to optimize the material and structural parameters to achieve it.

This could be accomplished by applying topology optimization (which uses different exact numerical procedures to optimize the layout or shape of a nanophotonic structure), adjoint optimization (calculates the sensitivity of a targeted result to variations in the nanophotonic structural parameters), stochastic optimization (e.g., iterated local search, stochastic gradient descent or stochastic hill climbing, where the design space is explored by random sampling of different configurations or introducing noise in the exploration procedure), Bayesian optimization (uses probabilistic models and combines prior knowledge with the observed data to sequentially optimize the nanophotonic design), convex optimization (includes least squares and linear programming; all constraints are convex functions, and the objective function is convex or concave if minimizing or maximizing, respectively), machine learning and neural networks (described earlier in this text), and last but not least, metaheuristic algorithms (such as evolutionary approaches, PSA, simulated annealing and ant colony optimization). An excellent and very detailed review of the approaches briefly summarized in this paragraph was published by Cai et al. in 2020 in *Nature Photonics* [259], which also presented a comparison between the conventional intuitive design of a metalens and the inverse design by evolutionary optimization. A review of equally high quality was published in the same journal by Molesky et al. [260], dealing with inverse design methods. A critical review of the use of deep learning for inverse design in nanophotonics, dealing both with the state of the art at the time and with perspectives and possible directions of that research that go “beyond inverse design”, was published by Wiecha et al. in the same year [261].

Somewhat unexpectedly, Barry et al. observed [262] that the application of evolutionary algorithms for the optimization of nanophotonic structures almost inevitably leads to the convergence of these artificially created forms to the corresponding natural structures. The authors remarked that both natural and artificial nanophotonic forms are fundamentally modular and that each building block has a role that can be understood almost independently from the rest of the complete structure. In their paper, this surprising similarity between the synthetic and natural was called “in silico evolution”.

Our primary goal here is to consider bio-inspired methods as tools for exploring the complex and high-dimensional design spaces encountered in nanophotonics. These may include both metaheuristic algorithms and artificial neural networks as well as other optimization techniques. The design spaces that need to be optimized are vast and include a large number of optimization parameters, and the optimization processes can be very demanding regarding computational time in case of complex problems. Some of the typical optimization goals in nanophotonics are listed in the further subsections.

### 10.1. Optimization of Parameters of Nanophotonic Materials

Nanomaterials for nanophotonics are, in no particular order and without claims for comprehensiveness, nanoparticles (including core-shell structures and quantum dots), 2D materials such as graphene or MXenes, atomically thin materials, and 1D materials such as nanowires, as well as nanocomposites such as multilayer ultrathin-film structures, plasmonic crystals, hypercrystals, meta-atoms and meta-molecules regarded as building blocks for metamaterials and many more. The basic materials, out of which the above have been designed, include conventional dielectrics, metals and semiconductors, encompassing, e.g., transparent conducting oxides (TCOs), liquid crystals, ferroelectrics, phase change materials and many more.

The nanomaterial effective parameters that can be optimized in a biomimetic manner include complex relative dielectric permittivity, complex relative magnetic permeability, spectral and spatial dispersions, spatial anisotropy, and nonlinear and quantum properties. Their optimization can be used to enhance light-matter interactions, tailor spectral responses (transmissivity, reflectivity or absorptivity/emissivity), optimize the bandwidth of nanophotonic building blocks and devices, etc. In 2018, Peurifoy et al. considered the inverse design of multilayer photonic nanoparticles by training artificial neural networks [263]. Their nanoparticles were of the core-shell type with two to eight different shells, and their goal was to start with any desired spectral dispersion and to determine the nanoparticle geometry that reproduces that dispersion as closely as possible. Their results were more accurate for more difficult problems with a higher number of shells and with more parameters. The procedure they utilized required only a rather small number of samples for effective ANN training, and the processing times of their calculations were orders of magnitude shorter than those required for conventional numerical simulations. Liu, Maier and Li considered the optimization of meta-atoms for enhanced light absorption and coloration by metaheuristics (genetic algorithms) [264]. They extended the unit cell to encompass more than a single meta-atom. The situation they arrived at was that the problem became vastly more complex, but still solvable by a genetic algorithm. The gain from such an approach was that the performance of the investigated nanophotonic system was significantly enhanced compared to the single meta-atom unit cell, owing to a wider search space and better opportunities for optimization. In [197], the authors performed the optimization of high-refractive-index photonic material geometry using the differential evolution metaheuristic algorithm. They optimized the design of nanostructured planar Si dielectric antennas coupled to a Gd_2_O_3_ nano-matrix doped with Eu^3+^ emitters in order to control their emission rate. For this purpose, they searched for global extrema of the decay rate enhancements (Purcell factor) of the magnetic and electric dipolar transitions of their structure. A hybrid multi-objective optimization method has been used to simultaneously optimize materials and dimensions of multilayer nanophotonic structures using Monte Carlo simulation with a continuous adaptive genetic algorithm and a pattern search algorithm [265]. The authors demonstrated their method by designing an ultra-broadband perfect absorber for visible and near-infrared wavelengths.

### 10.2. Optimization of Nanostructural Design of Basic Nanophotonic Building Blocks

The building blocks for nanophotonics encompass photonic bandgap materials (photonic crystals), metamaterials and their increasingly popular and widening subgroup of metasurfaces, as well as different kinds of nanoplasmonic structures including plasmonic crystals, extraordinary optical transmission (EOT) arrays and electromagnetically induced transparency (EIT) structures. The boundaries between nanophotonic building blocks and nanophotonic materials described in the previous subsections are blurred, and they sometimes overlap, especially in the case of artificial nanocomposite materials.

Each of the above nanophotonic building blocks branches into a multitude of different solutions. For instance, metamaterials and metasurfaces are further subdivided (some of the subgroups may overlap) into left-handed materials, zero-index metamaterials, artificial dielectrics, very-high-index metamaterials, Huygens’ metamaterials, metasurface holograms, plasmonic metamaterials, digital metamaterials, intelligent metasurfaces, coding metamaterials, reprogrammable metamaterials, time-varying (temporal-modulation) metamaterials, etc.

Bio-inspired procedures such as neural networks or metaheuristic algorithms can perform searches in design spaces for the optimal geometries, materials and arrangements of the building blocks to achieve desired optical properties, including light confinement, dispersion control, enhanced light-matter interactions, and tailored transmission or reflection characteristics.

Elsawy et al. [266] specifically considered the numerical optimization of metasurfaces. They mentioned both direct and inverse design methodologies. Of metaheuristic algorithms, they reviewed not only genetic algorithms and particle swarm optimization, but also hybrid approaches such as the covariance matrix adaptation evolution strategy. A part of their consideration was dedicated to deep learning using artificial neural networks. Other optimization methodologies, non-bio-inspired, were also analyzed, such as gradient-based design, including topology optimization.

In their mini-review, Qiu et al. [267] described the perspectives of the future developments of metasurfaces within the context of machine learning. Abdelraouf et al. [268] surveyed the latest advances in tunable metasurfaces, with a particular emphasis on AI-based design and optimization methodologies, mostly machine learning and, within that, mostly deep learning using artificial neural networks.

Li et al. [269] systematically reviewed intelligent metasurfaces, their applications and their design using AI methods (namely deep learning using artificial neural networks). They define intelligent metasurfaces as “smart platforms to manipulate the wave-information-matter interactions” and consider their control using ANN.

A machine learning-based multi-objective metasurface design for high-efficiency optimization of thermal emitters using a merging of topology optimization with generative adversarial networks (adversarial autoencoders) was presented in [270] by Kudyshev, Kildishev, Shalaev and Boltasseva. The authors reported an impressive 4900 times higher optimization speed, proving the power of their AI-based hybrid approach.

### 10.3. Optimization of Nanophotonic Devices

Nanophotonic devices are very diverse and cover many different fields of nanooptics. They include affinity-based plasmonic chemical, biochemical or biological sensors; light receptors such as nanophotonic photodetectors, including nanostructured solar cells (e.g., plasmonic solar cells, nanostructured photovoltaics, etc.); nanoantennas; light sources such as nanolasers, spasers (plasmonic lasers), quantum dot lasers and nanostructured LED diodes; super-resolution microscopes (e.g., stimulated emission depletion (STED), structured illumination microscopes (SIMs), spectral precision distance microscopes (SPDMs)); in-chip optical interferometers; and many more. More exotic nanophotonic devices include for instance superabsorbers, vector vortex beam generators, metalenses, hyperlenses, polarization converters and metasurface holograms.

Parameters in search spaces for nanophotonic devices are their material properties: complex relative index (real part, imaginary part (absorption coefficient or gain) and spectral dispersion characteristics); the geometry: dimensions, shapes, and arrangements of nanostructures, such as nanowires, nanoparticles, meta-atoms or metamolecules, 2D materials and plasmonic building blocks; and their polarization parameters. In case a device includes one or more optical waveguides, the targeted parameters may include waveguide geometry (width, height, length and shape) and such waveguide optical parameters as the refractive index contrast. If the device includes diffraction gratings, the targeted parameters may include the grating period, duty cycle and shape.

Metaheuristic algorithms and ANN can perform a search in design space and optimization for a number of desired optical properties of nanophotonic devices. A list may include transmission and reflection spectral dispersion characteristics (dispersion management), absorption efficiency (maximizing light absorption in specific regions of the spectrum for applications such as superabsorbers, photodetectors and solar cells), waveguiding efficiency (e.g., achieving low-loss propagation), resonance behavior (attaining desired resonant modes/resonant frequencies), nonlinear effects (optimizing, e.g., second harmonic generation, four-wave mixing or optical switching), quantum effects, directionality (controlling the directionality of light emission or scattering) and enhanced light-matter interactions.

The paper [177] described a hybrid between two metaheuristic algorithms, particle swarm optimization and gravitational search, for the optimization of the ultimate efficiency of silicon nanowire-based solar cells. The numerical simulations of the structures were performed using the finite-difference time-domain method. The final result of metaheuristic optimization was an ultimate efficiency of 42.5%, vastly exceeding the Shockley-Queisser limit for single p-n junction photovoltaics.

In their *Nature Communications* article, Nugroho et al. [271] reported the use of particle swarm optimization for the inverse design of hydrogen sensors based on plasmonic metasurfaces consisting of ordered palladium nanoparticle arrays. These sensors show sub-second speeds and are based on the intercalation of hydrogen atoms within the palladium crystal lattice. The authors fabricated their metasurface sensors and achieved record sensitivities of the order of parts per billion.

Differential evolution optimization of silicon-based dielectric nanoantennas ensuring the emission rate control of magnetic or electric dipolar emitters was performed in [197]. In that paper, the mentioned metaheuristic evolutionary algorithm was coupled to the green dyadic method and finite element method simulations. Other publications dedicated to evolutionary optimization include the design of plasmonic directional antennas by Wiecha et al. [272] and the design of all-dielectric magnetic nanoantennas by Bonod et al. [273].

**Figure 29:**
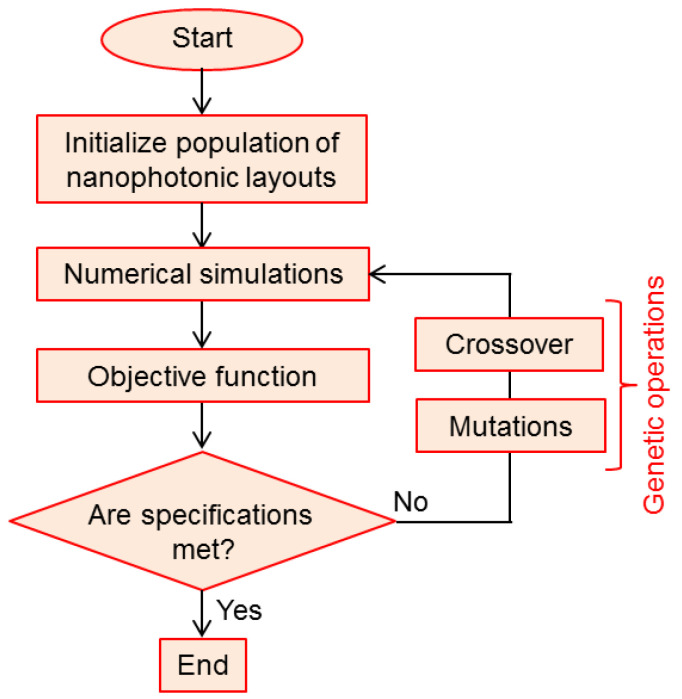
Simplified flowchart of the optimized design of a metasurface-based flat metalens. Based on [259].

A biomimetic procedure based on an evolutionary algorithm for the optimization of a metasurface-based flat metalens was described in [259]. Figure 29 shows a simplified flowchart of the optimized design process.

The use of artificial neural networks for the design of integrated photonic devices was investigated by Hammond and Camacho [274]. Among other things, they considered as a case study ANN-assisted design of the photonic circuit building blocks such as strip waveguides and chirped Bragg gratings.

### 10.4. Optical Waveguide Optimization

Metaheuristic algorithms can perform searches in design spaces for the optimal design of optical waveguides and their parameters. They can optimize their geometry, dimensions, shapes of elements and spatial profiles of optical constants (e.g., refractive index). The objectives can be optimization of modal confinement, minimization of wave propagation losses and thus maximization of light transmission through the optical guide, improvement of mode matching, etc. Among the main advantages of metaheuristic algorithms in nanophotonic design is the possibility to adapt and tailor them to specific highly complex problems and their applicability to very large search spaces with many parameters and constraints. Metaheuristic algorithms are also convenient for multi-objective optimization in nanophotonics in the case of multitudinous conflicting objectives, such as maximizing light extraction while minimizing scattering losses or achieving high-quality factor resonances while maximizing bandwidth. Multi-objective metaheuristics applied to nanophotonics can ensure the best trade-off solutions on the Pareto front.

Shiratori et al., from the group of Prof. Toshihiko Baba, reported the use of particle swarm optimization for the design of photonic crystal waveguides for slow light coupling with photonic integrated circuits [275]. PSO-based engineering of the arrangement of holes in 2D photonic crystals ensured drastically reduced experimental coupling losses of 0.21 dB (more than twice lower in comparison to dedicated tapered couplers), while the theoretically achievable losses were 0.12 dB on average in the telecommunication wavelength range of 1540–1560 nm. The optical waveguide design by way of ANN was also considered in [274].

### 10.5. Optimization of Photonic Circuit Design

A bio-inspired approach can be applied to optimize the photonic circuit element properties and the layout and routing of photonic circuits. It can optimize the spatial arrangement of optical components, including optical waveguides, beam splitters, couplers and modulators, to minimize losses and photonic circuit footprint and maximize signal quality. The routing of optical interconnects can be optimized to avoid or minimize crosstalk and interference. Multi-objective requirements can be also achieved, such as maximizing light extraction while at the same time minimizing scattering losses. Photonic circuits can be optimized in the sense of minimizing their sensitivity to variations or fluctuations of their parameters caused by the design itself, by the choice of the building materials and their properties, or by fabrication technology-induced uncertainties and geometrical “noises” [276], as well as the sensitivity to real operating conditions and signal fluctuations. In this way, improved robustness of photonic circuits to parameter fluctuations can be obtained.

The article by Dinc et al. [277] investigated the use of deep neural networks for the design of photonic circuits. The emphasis was on novel 3D all-optical circuits because of their promise for scalability. The authors considered transitions from 2D (data input) to 3D (circuit) and back. To do so, they started from the knowledge base of tomography techniques and used their conclusions.

In their *Nature Photonics* paper Shen et al. [278] described and experimentally demonstrated the implementation of artificial neural network functionality by way of a silicon photonic integrated circuit consisting of a cascaded array of 56 programmable Mach-Zehnder interferometers. They demonstrated the usability of the all-optical chip-based deep-learning ANN for vowel recognition. Thus, instead of using AI to optimize the photonic circuit, they used a photonic circuit as a hardware implementation of AI.

A recent review of works investigating the use of all-optical computing for artificial intelligence applications has been published in *Nature* by Wetzstein et al. [279]. The paper discussed the promises of this approach, as well as its challenges. A hardware implementation of an all-optical diffractive deep neural network was presented in *Science* in the article by Lin et al. [280]. In it, various applications of the proposed all-optical neural network in feature detection, image analysis, advanced camera design and object classification were described.

### 10.6. Future Directions

Challenges that define future directions of bio-inspired optimization algorithms in research and practical implementation in nanophotonics include the general development of multi-objective optimization and hybrid optimization methods (see Section 6 and Section 7), the integration of multiphysics into nanophotonics optimization (problems of coupling with e.g., thermal or quantum phenomena), and further development of solutions of inverse design problems, especially the highly complex ones. Another direction of future research is related to biomimetic nanophotonics, where the best performance is expected from nanophotonic systems mimicking and even upgrading the natural designs. As previously mentioned in this section, some AI-optimized nanophotonic systems already spontaneously exhibit biology-like features.

Future developments are also likely to include introducing real-world experimental constraints into biomimetic optimization. We expect that this should bring optimized practical implementations to a completely new level. One of the directions of future research is surely the all-optical hardware integration of optimization algorithms, i.e., the development of dedicated optical integrated circuitry for facilitated and accelerated implementation of complex optimization procedures. As mentioned previously in this section, this research field is already starting to flourish. Ultimately, biomimetically AI-optimized breakthroughs in both the design and the application of nanophotonics are expected to revolutionize the whole field.

## 11. Discussion

In this part of our review, we discuss and summarize some items of interest for the practical implementation of biomimetic optimization algorithms. We also add some general considerations of the algorithms. However, it is not only impractical but literally impossible to consider each of the quoted algorithms separately. The number of possible combinations within, e.g., hybrid methods is vast, so we could not possibly cover each and every one of them. A similar consideration is valid for hyper-heuristics. Thus, we limit ourselves mostly to metaheuristics and neural networks, while the hybrid methods and hyper-heuristics are covered globally, each one of them being considered as a single item. Another warning is related to the “No Free Lunch” theorem [76]—there is no global rule on the suitability of optimization algorithms. Our own experience is that some approaches may prove excellent in one type of problem and very poor in another, sometimes unexpectedly so. One such example out of many was our optimization of an analog preamplifier circuit area for early epileptic seizure detection which was designed better by applying an ANN-assisted goal attainment method than by attempting to independently apply several well-known metaheuristic algorithms [231]. Useful tools regarding this problem are the existing sets of benchmark tests which help uncover some advantages and disadvantages of algorithms, as well as the publications that give results of their applications [81,82,83,97]. Even so, a lot of trial and error work, skill and effort may still be necessary.

### 11.1. Comparative Advantages and Disadvantages of Selected Optimization Algorithms 

Awareness of the advantages and disadvantages of bio-inspired optimization algorithms could be useful to the readers in further understanding the situations and contexts in which a chosen method would be optimally utilized. For a more profound insight into the criteria of the choice of the most convenient algorithm, we direct the reader to the above-quoted papers on algorithm benchmarking.

A caveat is due here: although bio-inspired algorithms generally are known for their effectiveness in solving complex optimization problems, sometimes they may be outperformed by traditional optimization algorithms, such as gradient-based optimization methods. Because of that, to find the most suitable optimization algorithm, it is of crucial importance to thoroughly assess the type of problem to be solved, to consider the characteristics of the search space and to take into consideration the available computing resources.

In the following part of this paper, we present our view on the most important advantages and disadvantages of different algorithms listed in the prior sections. The presented data are valid for the algorithms in their basic form, not for enhanced, improved or otherwise modified versions. We stress that hyper-heuristics and general hybrid methods are each treated as a separate group. We give the pertinent information in tabular form since it is the clearest and most evident way to present such data. These are general properties only (Table 9, Table 10, Table 11, Table 12, Table 13, Table 14, Table 15 and Table 16), and the concrete suitability of a particular algorithm will depend on the specific problem characteristics, parameter settings and concrete implementation.

The above data are by no means exhaustive and are more of an illustrative nature, being included for the sake of completeness. The readers are advised to consult dedicated analyses in the literature. For instance, in the case of metaheuristics, a good start could be to consult the review of Ma et al. from 2023 [97] for additional information (it does not cover the same aspects as the present paper and even completely skips some of its subjects, but does an outstanding job with metaheuristics and their modifications, improvements and hybrids).

### 11.2. Comparative Computational Costs of Selected Optimization Algorithms 

Among the most important criteria for selecting a particular optimization algorithm are surely its running speed and general computational costs. Again, the same warnings to users are necessary—there are a lot of modifications of the basic optimization algorithms which may exhibit totally different computational costs although stemming from the identical root. In addition, hybridizing algorithms or creating hyper-heuristics results in totally different computational environments with vastly changed properties, and the differences may be dramatic. Below, we consider only the basic algorithms, out of sheer impossibility to cover each and every one of the countless special cases and modifications or combinations. This time, we present a single table for all the algorithms under consideration. A qualitative partial overview is shown in Table 17. We take into account the time complexity (running time), memory efficiency, possibilities to parallelize computations and to upscale them to more complex search spaces and higher number of variables, and convergence and accuracy of the algorithms. A warning to the readers is due here: the properties shown in Table 17 are valid only for the basic forms of algorithms and represent qualitative and averaged estimations only and should be approached with caution. The real computational properties may vary widely in dependence on a particular problem and its details, as well as on whether a particular algorithm is modified or upgraded.

## 12. Conclusions

In this article, we attempted to offer a broad systematic survey of bio-inspired optimization methods, including the most recent information and the latest developments. We presented a modified taxonomy of bio-inspired algorithms and gave short descriptions of some prominent methods. We stressed selected heuristic, metaheuristic and hyper-heuristic approaches, their inclusion being based on the choice of the most often published and cited methods. Besides those, we also considered bio-inspired approaches such as ANNs that are unrelated to the heuristic methods, yet are often used in conjunction with them. We took care to include some newly proposed methods, the most recent ones among them being less than a year old, the rule for their inclusion being their citation numbers and their perceived importance. We also handled approaches such as hybridization of algorithms and multi-objective analysis. We focused on two interrelated fields that are seldom considered together in this kind of review, namely microelectronics and nanophotonics. For microelectronic circuits, we considered the optimization of parameters of passive and active circuit elements, floor planning and topology, circuit sizing, power consumption minimization, sensitivity to design parameter variations/robustness, production yield, and the applicability to analog, digital and mixed-type microelectronic circuitry. For nanophotonics, we considered the optimization of nanocomposite materials of interest, the design and optimization of their effective optical parameters, inverse design of fundamental nanophotonic building blocks such as photonic crystals, plasmonic structures, metamaterials including metasurfaces, photonic circuit design including layout and routing, analysis of sensitivity to parameter variations, and related robustness of design of photonic circuits and design of optical waveguides. We attempted to profile our treatise towards experienced researchers in the field who want to stay informed about the latest developments, to beginners and to interested scientific readers outside the field. 

Regarding the limitations of biomimetic optimization, they partly stem from the “No Free Lunch” theorem. High skills but also guesswork are still often necessary to choose the procedures/hybrids that are right for a specific problem. A difficulty also arises from the huge number of existing metaheuristic algorithms, which is still increasing even now, although at a slower pace, making the choice even more problematic. Depending on a particular algorithm, the limitations may also include slow convergence, high computational costs, especially when scaling up the search space to higher dimensionality, premature convergence either due to becoming stuck in a local optimum instead of finding the global or ineffectively exploring the search space, rather complex implementation (especially in case of multi-objective optimization), cumbersome parameter tuning, “black box” operation (lack of insight into the details of optimization process), inability to perform optimization without domain-specific data, and sensitivity to noisy or discrete datasets (lack of robustness).

Regarding the future prospects, we expect that the number of newly proposed metaheuristics will decrease, while journals will continue their trend of imposing more and more stringent conditions on new proposals of metaphor-based algorithms. In the authors’ opinion, chances for a drastic modification of a metaheuristic classification that would introduce some order and clearer criteria of algorithm novelty are currently slim, although this would be highly beneficial. The scientific community is too dependent on the customary metaphor-based classification, in spite of its obvious shortcomings. On the other hand, we expect large and significant developments in the fields of multi-objective procedures, and even more so in the field of hybrid solutions. We also expect that novel procedures will become more sophisticated, complex, transparent (with enhanced interpretability of the solutions) and general—able to solve optimization problems in a wider range of problems simultaneously, including noisy or dynamically changing environments. Another direction where we anticipate large advances is concrete, real-world applications which we believe will become more numerous and used in an even wider circle of problems and different fields. Finally, in some instance not far from today, we see the cumbersome process of choosing and implementing an adequate and fully customized optimization procedure performed automatically, thus making room for using human efforts in a more creative way. AI procedures in general will continue introducing a silent revolution in the field of biomimetic optimization, in a manner similar to that in which they are acting in uncountable fields at this very moment.

## Figures and Tables

**Table 1:** Selected heuristic algorithms, excluding metaheuristics or hyper-heuristics.

Algorithm Name	The Main Properties of the Algorithm	Ref.
Divide and Conquer Algorithm	The problem is decomposed into smaller, manageable sub-problems that are first independently solved in an approximate manner and then merged into the final solution.	[84]
Hill Climbing	The algorithm explores the neighboring solutions and picks those with the best properties, so that the algorithm constantly “climbs” toward them.	[85]
Greedy Algorithms	Immediate local improvements are prioritized without taking into account the effect on global optimization. The underlying assumption is that such “greedy” choices will result in an acceptable approximation.	[86]
Approximation Algorithms	Solutions are searched for within provable limits around the optimal solution. The aim is to achieve the maximum efficiency. This is convenient for difficult nondeterministic polynomial time problems.	[87]
Local Search Algorithms	An initial solution is assumed, and it is iteratively improved by exploring the immediate vicinity and making small local modifications. No completely new solutions are constructed.	[88]
Constructive Algorithms	Solutions are built part-by-part from an empty set by adding one building block at a time. The procedure is iterative and uses heuristics for the choice of the building blocks.	[89]
Constraint Satisfaction Algorithms	A set of constraints is defined at the beginning. The solution space is then searched locally, each time applying the constraints until all of them are satisfied.	[90]
Branch And Bound Algorithm	The solution space is systematically divided into smaller sub-problems, the search space is bounded according to problem-specific criteria, and branches that result in suboptimal solutions are pruned and removed.	[91]
Cutting Plane Algorithm	An optimization method solving linear programming problems. It finds the optimal solution by iteratively adding new, additional constraints (cutting planes), thus gradually tightening the region of possible solutions and converging towards the optimum.	[92]
Iterative Improvement Algorithms	Here the goal is to iteratively improve an initially proposed problem solution. Thus, systematic adjustments and improvements are made to the initial set by targeting the predefined objectives. The values may be reordered, retuned or swapped until the desired optimization is complete.	[93]

**Table 2:** Selected evolutionary algorithms.

Algorithm Name	Abbr.	Proposed by, Year	Ref.
Genetic Algorithm	GA	Holland, 1975	[99]
Memetic Algorithm	MA	Moscato, 1989	[100]
Differential Evolution	DE	Storn, 1995	[101]

**Table 3:** Selected swarm intelligence algorithms.

Algorithm Name	Abbr.	Proposed by, Year	Ref.
Particle Swarm Optimization	PSO	Eberhart, Kennedy, 1995	[104]
Whale Optimization Algorithm	WOA	Mirjalili, Lewis, 2016	[105]
Gray Wolf Optimizer	GWO	Mirjalili, Mirjalili, and Lewis, 2014	[106]
Artificial Bee Colony Algorithm	ABCA	Karaboga, 2005	[107]
Ant Colony Optimization	ACO	Dorigo, 1992	[108]
Artificial Fish Swarm Algorithm	AFSA	Li, Qian, 2003	[109]
Firefly Algorithm	FA	Yang, 2009	[110]
Fruit Fly Optimization Algorithm	FFOA	Pan, 2012	[111]
Cuckoo Search Algorithm	CS	Yang and Deb, 2009	[112]
Bat Algorithm	BA	Yang, 2010	[113]
Bacterial Foraging	BFA	Passino, 2002	[114]
Social Spider Optimization	SSO	Kaveh et al., 2013	[115]
Locust Search Algorithm	LS	Cuevas et al., 2015	[116]
Symbiotic Organisms Search	SOS	Cheng and Prayogo, 2014	[117]
Moth-Flame Optimization	MFOA	Mirjalili, 2015	[118]
Honey Badger Algorithm	HBA	Hashim et al., 2022	[119]
Elephant Herding Optimization	EHO	Wang, Deb, Coleho, 2015	[120]
Grasshopper Algorithm	GOA	Saremi, Mirjalili, Lewis, 2017	[121]
Harris Hawks Optimization	HHO	Heidari et al., 2019	[122]
Orca Predation Algorithm	OPA	Jiang, Wu, Zhu, Zhang, 2022	[123]
Starling Murmuration Optimizer	SMO	Zamani, Nadimi-Shahraki, Gandomi, 2022	[124]
Serval Optimization Algorithm	SOA	Dehghani, Trojovský, 2022	[125]
Coral Reefs Optimization Algorithm	CROA	Salcedo-Sanz et al., 2014	[126]
Krill Herd Algorithm	KH	Gandomi, Alavi, 2012	[127]
Gazelle Optimization Algorithm	GOA	Agushaka, Ezugwu, Abualigah, 2023	[128]

**Table 4:** Selected algorithms mimicking human or zoological physiological functions.

Algorithm Name	Abbr.	Proposed by, Year	Ref.
Artificial Immune System	AIS	Dasgupta, Ji, Gonzalez, 2003	[142]
Neural Network Algorithm	NNA	Sadollah, Sayyaadi, and Yadav, 2018	[143]
Human Mental Search	HMS	Mousavirad, Ebrahimpour-Komleh, 2017	[144]

**Table 5:** Selected anthropological algorithms (mimicking human social behavior).

Algorithm Name	Abbr.	Proposed by, Year	Ref.
Imperialist Competitive Algorithm	ICA	Atashpaz-Gargari et al., 2007	[148]
Anarchic Society Optimization	ASO	Ahmadi-Javid, 2012	[149]
Teaching-Learning Base Optimization	TLBO	Rao, Savsani, and Vakharia, 2011	[150]
Society and Civilization Optimization	SC	Ray et al., 2003	[151]
League Championship Algorithm	LCA	Kashan, 2009	[152]
Volleyball Premier League Algorithm	VPL	Moghdani, Salimifard, 2018	[153]
Duelist Algorithm	DA	Biyanto et al., 2016	[154]
Tabu Search	TS	Glover, Laguna, 1986	[155]
Human Urbanization Algorithm	HUA	Ghasemian, Ghasemian, Vahdat-Nejad, 2020	[156]
Political Optimizer	PO	Askari, Younas, Saeed, 2020	[157]

**Table 6:** Selected plant-based algorithms.

Algorithm Name	Abbr.	Proposed by, Year	Ref.
Flower Pollination Algorithm	FPA	Yang, 2012	[159]
Invasive Weed Optimization	IWO	Mehrabian, Lucas, 2006	[160]
Plant Propagation Algorithm	PPA	Salhi, Fraga, 2011	[161]
Plant Growth Optimization	PGO	Cai, Yang, Chen, 2008	[162]
Tree Seed Algorithm	TSA	Kiran, 2015	[163]
Paddy Field Algorithm	PFA	Premaratne, Samarabandu, Sidhu, 2009	[164]

**Table 7:** Bio-inspired multi-objective optimization algorithms and selected examples of their applications in microelectronics and photonics including sensorics and fog/cloud computing.

Algorithm Name	Some Applications, References
Multi-Objective (MO) Genetic Algorithm	Improvement of photoelectric performance of thin film solar cells [184] Optimization of nanosecond laser processing [185] VLSI floor planning optimization regarding measures such as area, wire length and dead space between modules [186] Lifetime reliability, performance and power consumption of heterogeneous multiprocessor embedded systems [187]
MO Particle Swarm Optimization	Review of many applications of MO PSO in diverse areas [188] Floor planning of the VLSI circuit and layout area minimization using MO PSO [189]
MO Ant Colony Optimization	A 3D printed bandpass frequency-selective surface structure with desired center frequency and bandwidth [190] Analog filter design [191] Multi-criteria optimization for VLSI floor planning [192]
Artificial Bee Colony	Area and power optimization for logic circuit design [193] Design of digital filters [194]
Artificial Immune System	Spectrum management and design of 6G networks [195] Multi-objective design of an inductor for a DC-DC buck converter [196]
Differential Evolution	Geometry optimization of high-index dielectric nanostructures [197] Multi-objective synchronous modeling and optimal solving of an analog IC [198]
Firefly Algorithm	Reducing heat generation, sizing and interconnect length for VLSI floor planning [199] Secure routing for fog-based wireless sensor networks [200]
Cuckoo Search	Multi-objective-derived energy-efficient routing in wireless sensor networks [201] Parameter extraction of photovoltaic cell based on a multi-objective approach [202]
MO Grey Wolf Optimizer	Electrochemical micro-drilling in MEMS [203] Multi-objective task scheduling in cloud-fog computing [204]

**Table 8:** ANNs within the context of bio-inspired optimization algorithms.

Metaheuristic optimization algorithms (e.g., genetic algorithms (GAs), ant colony optimization (ACO), differential evolution (DE) and particle swarm optimization (PSO)) can be used for the optimization of ANN parameters.
ANNs themselves can serve as optimization algorithms (Hopfield networks, Boltzmann machines).
ANNs can generate efficient approximations of complex objective functions subjected to optimization within metaheuristics.
They can be used for hyperparameter optimization of other machine learning algorithms.
They ensure extraction or simultaneous feature extraction with hyperparameter optimization

**Table 9:** Advantages and disadvantages of evolutionary algorithms.

Algorithm	Advantages	Disadvantages
Genetic Algorithm (GA)	convenient for a wide range of problems good for both discrete and continuous variables global search capabilities enables exploration of a diverse search space	high computational cost computational cost scales with problem complexity often needs additional techniques to handle constraints
Memetic Algorithm (MA)	combines global search with local search (meme) adaptable and customizable can incorporate problem-specific knowledge effective constraint handling fast convergence due to enhanced local search	complex algorithm non-trivial and problem-specific design difficult parameter tuning computationally complex and demanding may become stuck in a local optimum
Differential Evolution (DE)	good for continuous data low algorithm complexity minimal number of control parameters for tuning usable for noisy and non-differentiable fitness functions robust user-friendly	poor for discrete data performs worse with upscaled search spaces may become stuck in local optima lack of population diversity sensitive to control parameter tuning lower convergence speed

**Table 10:** Advantages and disadvantages of swarm intelligence algorithms.

Algorithm	Advantages	Disadvantages
Particle Swarm Optimization (PSO)	good for continuous data high efficiency at finding a single global optimum fast convergence for simple search spaces user-friendly	struggles with discrete data lack of population diversity may become stuck in a local optimum poor performance with complex search spaces
Ant Colony Optimization (ACO)	good for handling discrete data good exploration and exploitation convenient for combinatorial search spaces robust to problem changes	struggles with large-scale problems slow convergence may become stuck in a local optimum sensitive to parameter choice
Whale Optimization Algorithm (WOA)	good for global search simple and user-friendly handles both continuous and discrete problems low number of control parameters converges quickly in continuous search spaces	may become stuck in local optima in multimodal search spaces sensitive to parameter setting struggles with exploration in irregular search spaces struggles with large-scale problems
Grey Wolf Optimizer (GWO)	applicable to both discrete and continuous problems good global search capabilities fast convergence simple parameter tuning overall simplicity and user-friendliness	struggles with large-scale problems may become stuck in a local optimum limited exploration capabilities in irregular search spaces sensitive to parameter tuning
Firefly Optimization Algorithm (FOA)	good global search capabilities good for both continuous and discrete problems fast convergence in continuous search spaces good for large-scale problems (parallelizable) simple and user-friendly	limited exploration capabilities in irregular search spaces may become stuck in a local optimum (premature convergence) poor population diversity sensitive to parameter tuning
Bat Optimization Algorithm (BOA)	good balance between exploration and exploitation good for both continuous and discrete problems includes local search capabilities fast convergence in continuous search spaces simple and user-friendly	limited exploration capabilities in irregular search spaces may become stuck in a local optimum struggles with large-scale optimization problems (poor scalability) sensitive to parameter tuning
Orca Predation Algorithm (OPA)	•good exploration/exploitation balance •ability to find multiple global solutions in multimodal problems good for problems with constraints	may struggle with global search may become stuck in a local optimum •poor diversification •may be prone to localization errors
Starling Murmuration Optimizer (SMO)	•good exploration/exploitation balance •good diversity •good for finding global optima, bypassing local minima •applicable to diverse search spaces •fast convergence	struggles with large-scale optimization problems (poor scalability) computational cost increases with the number of dimensions •relatively complex

**Table 11:** Advantages and disadvantages of artificial immune system algorithm.

Algorithm	Advantages	Disadvantages
Artificial Immune System (AIS)	good for upscaled and complex optimization problems parallelizable good for finding global optima while avoiding becoming stuck in local optima able to learn from search space and self-improve accordingly robust to noisy environments ensures diversity of solutions good for dynamic problems and pattern recognition	can be computationally complex and expensive lack of solid theoretical foundation challenging understanding and analysis complex and time-consuming parameter tuning applicable to a relatively narrow range of problems

**Table 12:** Advantages and disadvantages of anthropological algorithms.

Algorithm	Advantages	Disadvantages
Tabu Search Algorithm (TSA)	good for both discrete and continuous optimization good balance between exploration and exploitation retains memory structure/tabu list, thus escaping local optima robust against noisy environments simple parameter tuning	requires additional memory for tabu list struggles with large-scale problems sensitive to parameter tuning sensitive to tabu list tenure choice problems with convergence in complex search spaces

**Table 13:** Advantages and disadvantages of plant-based algorithms.

Algorithm	Advantages	Disadvantages
Flower Pollination Algorithm (FPA)	a good balance between exploration and exploitation good global search capabilities good for both continuous and discrete optimization problems fast convergence, especially in smooth and continuous search spaces simple and user-friendly	limited exploration in problems with irregular search spaces poor diversity of the population struggles with large-scale optimization problems (poor scalability) may become stuck in local optima sensitive to tuning of control parameters

**Table 14:** Advantages and disadvantages of the class of hyper-heuristics.

Algorithm	Advantages	Disadvantages
Hyper-Heuristic Algorithms	a general-purpose class of optimization methods usable for a wide range of problems not dependent on the problem representation or domain-specific knowledge able to smartly adapt search strategies able to learn from previous experience and use the knowledge to further problems reduced need for manual interventions	can be very complex to use and require expert knowledge lack insight into the problem structure or domain-specific knowledge can be computationally demanding may be bested by problem-specific optimization algorithms lack a full adaptability to the problem at hand

**Table 15:** Advantages and disadvantages of the class of hybrid algorithms.

Algorithm	Advantages	Disadvantages
Hybrid Algorithms	a class of diverse algorithms, complementing strengths of its constitutive parts improved solution quality improved robustness and adaptability improved convergence speed ability to exploit problem-specific knowledge applicable to an extended range of problems	higher complexity compared to its constitutive algorithms design, implementation and result analysis require expert knowledge more complex tuning of parameters due to their increased number computational cost may be high tends to overfit data

**Table 16:** Advantages and disadvantages of neural networks for optimization.

Algorithm	Advantages	Disadvantages
Artificial Neural Networks (ANNs)	excel in optimizing problems involving nonlinear relationships among variables performance and problem optimization are optimized in an adaptable way through learning able to generalize data never before seen by them and use this for optimization convenient for handling huge amounts of data allow for massive parallelization of computing indispensable in accurately approximating functions with any arbitrary continuous relationship ∙	suffer from lack of transparency (black box nature) difficult tuning of hyperparameters (number of layers and nodes, learning rate, etc.) resource-intensive need huge amounts of data for accurate training computationally demanding prone to overfitting (may fail to generalize well for new datasets and search spaces)
Convolutional Neural Networks (CNNs)	well suited for image-related optimization tasks and sequential data with spatial hierarchies able to recognize patterns regardless of their position in an image (translation invariance) automatic recognition and extraction of features during optimization offers transfer learning (using training in one search space for another) lower risk of overfitting thanks to parameter sharing across spatial dimensions	need huge amounts of data for accurate training computationally demanding resource-intensive suffer from lack of transparency (black box nature) •minuscule perturbations of the input data can result in incorrect predictions vulnerable to adversarial attacks, which negatively reflects on security and robustness

**Table 17:** Computational advantages and disadvantages of bio-inspired optimization algorithms.

Algorithm	Time Complexity	Memory Efficiency	Parallelizability	Scalability	Convergence and Accuracy
Genetic Algorithm	Limited	Good	Good	Good	Moderate
Memetic Algorithm	Moderate	Good	Good	Good	Limited
Differential Evolution	Moderate	Good	Good	Moderate	Good
Particle Swarm Algorithm	Moderate	Good	Limited	Moderate	Moderate to good
Ant Colony Optimization	Moderate	Good	Limited	Good	Moderate to limited
Whale Optimization Algorithm	Moderate	Good	Limited	Good	Moderate to good
Grey Wolf Optimizer	Moderate	Good	Limited	Good	Moderate to good
Firefly Optimization Algorithm	Moderate	Good	Limited	Good	Moderate to good
Bat Optimization Algorithm	Moderate	Good	Limited	Good	Moderate to good
Orca Predation Algorithm	Moderate to good	Good	Limited to moderate	Good	Moderate
Starling Murmura tion Optimizer	Moderate	Moderate to good	Limited	Limited	Good
Artificial Immune System	Moderate	Good	Limited	Good	Moderate
Tabu Search Algorithm	Moderate	Good	Limited	Good	Moderate
Flower Pollination Algorithm	Good to moderate	Good	Limited	Good	Moderate
Hyper-Heuristics	Moderate	Good	Limited	Moderate to good	Moderate to good
Hybrid Algorithms	Moderate to good	Good to moderate	Limited to moderate	Moderate to good	Moderate to good
Artificial Neural Networks	Moderate to good	Moderate to good	Limited to moderate	Moderate to good	Moderate to good
Convolutional Neural Networks	Moderate	Moderate	Good	Good	Good

## Data Availability

Data sharing not applicable.
